# Selection of reference genes for flowering pathway analysis in the masting plants, *Celmisia lyallii* and *Chionochloa pallens*, under variable environmental conditions

**DOI:** 10.1038/s41598-019-45780-1

**Published:** 2019-07-05

**Authors:** Samarth, Paula E. Jameson

**Affiliations:** School of Biological Sciences, University of Canterbury, Christchurch, New Zealand

**Keywords:** Phenology, Plant molecular biology

## Abstract

Mast flowering is characterised by mass synchronised flowering at irregular intervals over a wide geographical area. An understanding of the molecular drivers of mast flowering requires expression analysis of key developmentally regulated gene(s). Reverse transcription-quantitative PCR is the gold standard technique used to assess expression of target gene(s) and to validate high-throughput sequencing data. Selection and validation of appropriate reference gene(s), used as normalisation factors in transcript abundance analysis, is an essential step to avoid ambiguous expression results. Eight candidate reference genes were assessed to select the best internal normalisation factors in naturally growing masting plants *Chionochloa pallens* and *Celmisia lyallii*. Statistical packages geNorm, Normfinder, BestKeeper, ΔC_t_ and RefFinder were used to determine the expression stability in plants translocated to different altitudes and sampled across the season. *GAPDH* and *PP2a* in *Celmisia* and *ExP* and *THP* in *Chionochloa* were found to be the best pairs of reference genes for normalisation of the gene expression data. Our study revealed environmentally-induced changes in reference gene expression, information that will be utilised as we investigate flowering phenology of masting plants under global climatic change.

## Introduction

Advances in sequencing have revolutionised the field of gene expression, particularly in non-model organisms^1–4^. The onset of next-generation technologies and the rapid decrease in per-base sequencing costs has boosted the use of massively parallel cDNA sequencing (RNA-seq)^5,6^. RNA-seq is now a cost-effective technique, enabling the acquisition of large amounts of transcriptomic data from different sources. RNA-seq can be used to determine the amount of differentially expressed transcripts, novel genes, transcription factors, alternatively spliced variants and Single Nucleotide Polymorphisms (SNPs), even in species that lack a reference genome^7^. *De novo* transcriptome assembly is particularly advantageous when studying ecological, biological, cellular and molecular processes in distantly related non-model plant species^4,8–12^.

However, the data generated in RNA-seq or microarray analysis requires an authentic tool for validation^13–16^. Reverse transcription quantitative polymerase chain reaction (RT-qPCR) is the gold-standard used by researchers to validate data obtained from protocols such as RNA-seq^17^. The sensitivity, precision, reproducibility and real-time progression of the PCR reaction allows it to be the most accurate and reliable resource for confirming expression analysis data obtained from sources such as microarrays and RNA-seq^18,19^. It allows monitoring of expression profiles and mRNA abundance levels across different samples concurrently^20^. Moreover, RT-qPCR can be used for the detection of low expressed transcripts which cannot be determined through high-throughput sequencing analysis^21^. It can provide either an absolute number of cDNA copies or a relative quantification of the desired transcript across samples^22,23^. In the latter case, the amount of expression of the target gene is compared with the expression of an internal reference gene to normalise the copy numbers in different samples^24,25^.

Strategies have been employed to accurately normalise the variation in different samples for reproducible and precise measurements including starting with an optimum amount of material, having an adequate number of biological replicates as well as appropriate technical replication. Good quality RNA, cDNA quality checks, no template and negative RT controls, an amplification factor of 2.0, qPCR efficiency ranging between 90–110%, proper controls for normalisation and statistical analysis are all also necessary^26^. To date, normalisation of the target genes across different samples has been a key step in minimising the variations produced in qPCR^27,28^. Normalisation involves the selection of appropriate control/reference genes, whose expression is compared to the gene of interest in the test and control samples. The reference genes, previously referred to as housekeeping genes, are genes selected because they are constitutively expressed among different tissues and cells in different conditions. The expression of an ideal reference gene is stable across tissues and remains unaffected by experimental treatments^29^. Such genes generally belong to basic cellular processes, primary metabolism, or are cell structure determinants. Thus, many traditional reference genes used in plant-based research include *actin* (*ACT*), *elongation factor 1α* (*EF1α*), *glyceraldehyde*-*3*-*phosphate dehydrogenase* (*GAPDH*), *eukaryotic initiation factor 4ε* (*eIF4ε*), *ubiquitin* (*UBQ*), and *18S ribosomal RNA* (*U18S*).

There are numerous reports of selection and validation of reference genes in model plant species including *Arabidopsis thaliana*^20^, *Oryza sativa*^30^, *Helianthus annuus*^31^, *Triticum aestivum*^32,33^, *Zea mays*^34^ and *Glycine max*^35^. The list also includes diverse non-model plant species including flax^36^, white clover^37^, bamboo^38^, peach^39^, *Mimulus*^40^, watermelon^41^, grape vine^42^, and lettuce^43^. However, in most of these cases the selection of suitable reference gene(s) was based on tightly controlled experimental conditions, whereas it has been reported that the expression of these gene(s) can vary in different tissues under different experimentally controlled conditions^26,44–48^. Selection of such biased reference genes can result in misinterpretation of the qPCR data and, consequently, output of misleading expression data. In recent years, researchers have stressed the importance of selection and proper validation of reference genes under natural environmental conditions as a mandatory step before normalisation against the expression of the gene of interest^27,42^. The necessity of proper validation increases in research involving non-model plant species where genomic data is unavailable.

**Figure 1:**
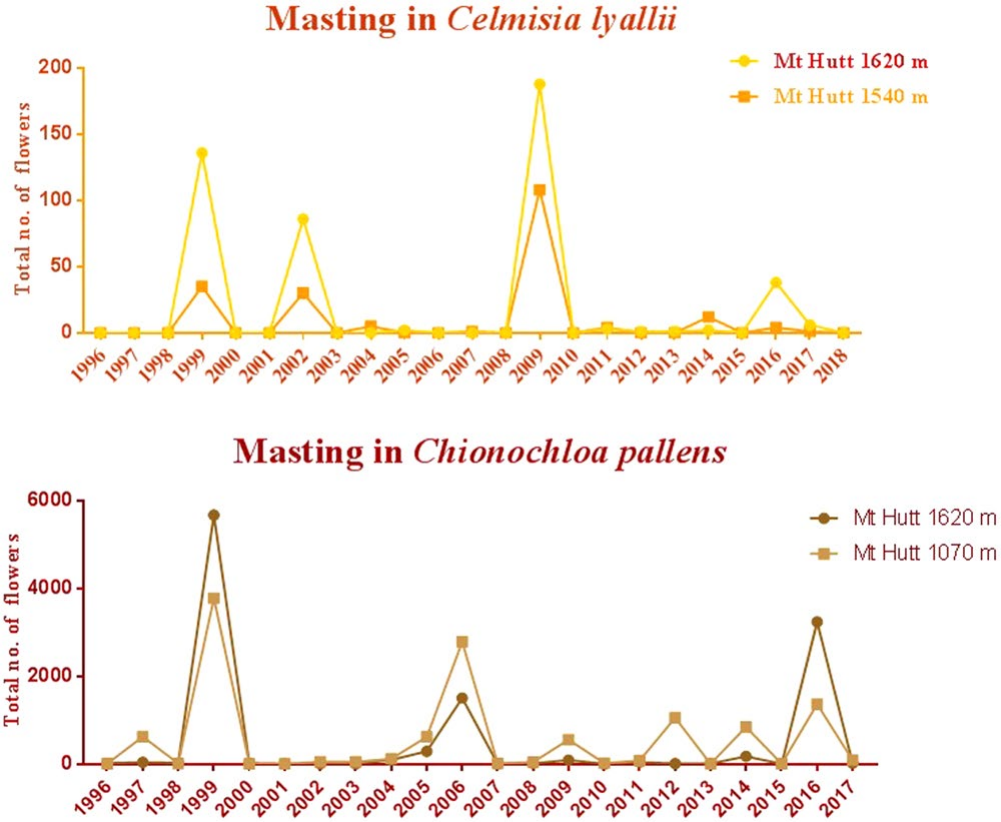
Masting in *Celmisia lyallii* and *Chionochloa pallens* using data collected from the Mt Hutt field site over the past 26 years. The y-axis corresponds to the number of flowers per plant, and the x-axis corresponds to the timescale represented in years.

This report deals with identification, selection and validation of suitable candidate reference genes for our study of the ecological phenomenon of masting in two non-model plant species, *Chionochloa pallens* (Poaceae) and *Celmisia lyallii* (Asteraceae)^49^. Masting is synchronised intermittent flowering and production of seeds by a perennial plant population over a wide geographical area^50,51^. *Chionochloa pallens* and *C*. *lyallii* are endemic to New Zealand and are the two strongest masting alpine plants (Fig. 1)^52^. Masting is a major problem in New Zealand. For example, *Nopthofagus* sp (beech) trees flower irregularly, typically once in every 2 to 6 years, resulting in the intermittent production of large quantities of seed. During the masting event, populations of seed predators, such as introduced rodents and stoats, increase dramatically. Due to the abundance of food, these invasive predators reproduce more frequently and their numbers rise rapidly. When the large populations of rodents and stoats have consumed the seeds, they turn to preying on indigenous species including kiwi, kaka, kakapo, kea and native bats. Rapid expansion of the rodent population can lead to an 80–90% decline in the population of mohua birds^53^. Such attacks are threatening the survival of endangered birds. Moreover, climate change may have effects at both an individual and at a population level in terms of the masting phenomenon. Consequently, it is crucial to identify the molecular markers regulating flowering-time control, so they can be used as predictive markers of masting in order to launch pre-emptive conservation measures.

It has been shown that the temperature difference between the two previous summers (ΔT) can be used to predict a masting season. The ΔT model is based on the seed fall measured in 40 different masting species over 26 years^52^. To induce mast flowering, we carried out translocation experiments in the field to provide, or not, an appropriate ΔT. We gathered samples for analysis of gene expression in order to gain an understanding of the molecular controls of mast flowering. Consequently, it became crucial to identify highly stable reference genes which remained unaffected in the diverse environmental conditions of our experiment. Improper identification and validation of the reference genes would lead to errors and misinterpretation of the gene expression data. Thus, it was critical to test and validate various reference genes prior to determining the molecular basis of flowering in these non-model plant species.

In this study, novel and potentially stable genes were identified from the *de novo* transcriptome assembly^54^ of *Chionochloa* and *Celmisia*. These genes were analysed based on the statistical programs geNorm^55^, Normfinder^56^, BestKeeper^57^, ΔC_t_^58^ and RefFinder^59^ in order to identify and rank the best possible reference gene(s) or combination of genes for the normalisation of the qPCR data obtained from the field experiments. Potential reference genes were tested against altitudinal translocations, and changes over season and development in leaves from vegetative and flowering plants. These parameters acted as strong controls for an unbiased identification of appropriate reference genes for further gene expression analysis.

## Results

A list of reference gene sequences was prepared based on previous reports of reference gene selection for plants in either the Asteraceae or Poaceae. Gene sequences encoding *elongation factor* 1-α (*EF*-*1α*)^60^, *glyceraldehyde*-*6*-*phosphate dehydrogenase* (*GAPDH*)^20^, *eukaryotic initiation factor 4ε* (*eIF*-*4ε*)^34^, *phosphoglycerate kinase 1* (*PGK1*)^61^, *protein phosphatase 2a* (*PP2a*)^61^, *metalloprotease* (*MTP*)^61^, *SAND*^61^, *actin* (*ACT*)^62^, *β*-*tubulin* (*TUB*)^30^, *expressed protein* (*ExP*)^63^, *nuclease*-*binding protein* (*NBP*)^63^, and *tumour homolog protein* (*THP*)^63^ were downloaded from the Genbank database and used to search for the corresponding homologous sequences in the *Celmisia* and *Chionochloa* transcriptomes (Table 1). Candidate reference gene sequences were identified from the transcriptomic data (unpublished) using TBLASTN^64^. Since most of the gene sequences belonged to a protein family, the identified candidate reference gene sequences were subjected to a phylogenetic analysis using MEGA 7.0^65^ to confirm their identity (Supplementary Data S1). All the putative reference gene sequences clustered with their corresponding protein family.

**Table 1:** Identification of the candidate reference genes from the *de novo* transcriptome assembly of *Celmisia* and *Chionochloa*.

Gene	Gene name	Homolog identifier	GenBank ID	Protein ID	% Identity	E-value	Reference
**Identification of candidate reference genes in** ***Celmisia***							
*C*. *lyallii EF1α*	*Elongation factor1α*	*Ha EF1α*	XM_022150167	XP_022005859	87.21	1.43E − 52	^63^
*C*. *lyallii eIF4ε*	*Eukaryotic inititaion factor 4ε*	*Ha eIF4ε*	HQ430514	AEB36860	83.65	8.66E − 98	^34^
*C*. *lyallii GAPDH*	*Glyceraldehyde*-*6*-*phosphate dehydrogenase*	*Ha GAPDH*	EU112608	ABW89100	89.29	0	^18^
*C*. *lyallii SAND*	*SAND family protein*	*Chs SAND*	KF752605	AHC13232	81.77	0	^64^
*C*. *lyallii MTP*	*Metalloprotease*	*Chs MTP*	KJ524574	AJF20615	78.5	0	^64^
*C*. *lyallii PGK1*	*Phosphoglyceratekinase1*	*Chs PGK1*	KJ524576	AJF20617	84.6	0	^64^
*C*. *lyallii PP2A*	*Protein phosphatase 2A*	*Ha PP2A*	XM_022138979	XP_021994671	82.8	0	^64^
**Identification of candidate reference genes in** ***Chionochloa***							
*C*. *pallens EF1α*	*Elongation factor 1α*	*Osa Elongation factor 1α*	AK061464	BAG87945	83.2	0.00E + 00	^63^
*C*. *pallens GAPDH*	*Glyceraldehyde*-*6*-*phosphate dehydrogenase*	*Osa Glyceraldehyde*-*6*-*phosphate dehydrogenase*	AK064960	BAG89296	87.4	0.00E + 00	^18^
*C*. *pallens NBP*	*Nucleic acid binding protein*	*Osa Nucleic acid binding protein*	LOC_Os06g11170	XP_015644323	87.76	1.47E − 84	^66^
*C*. *pallens ExP*	*Expressed protein*	*Osa Expressed protein*	LOC_Os07g02340.1	XP_015646888	85.71	6.24E − 37	^66^
*C*. *pallens THP*	*Tumour homolog protein*	*Osa Tumour homolog protein*	XM_015761574	XP_015617060	77.31	3.56E − 46	^66^
*C*. *pallens ACT*	*Actin 2*	*Zm ACT 2*	J01238	AAA33433.1	76.3	0.00E + 00	^65^
*C*. *pallens β*-*TUB*	*β*-*Tubulin*	*Zm tubulin β*-*2*	NM_001111956	NP_001105426	97.71	0	^30^

### Primer specificity and amplification efficiency

All the designed primers for the corresponding selected reference genes with their abbreviations, primer melting temperature (Tm), amplicon length, amplification efficiency and correlation coefficient are provided in Table 2. For *18S ribosomal RNA* (*U18S*), universal primers were used for ribosomal gene quantification. All the primers were tested for amplification efficiency using the LinRegPCR software. The software allows estimation of amplification efficiencies for individual samples for a single gene primer pair. The mean amplification efficiency ranged from 95.4 to 110.6. The coefficient of correlation for the primers ranged from 0.998 to 0.9999. Additionally, the designed primers were validated for specificity from the melt curve analysis. All the primers showed a single peak in the dissociation curve indicating the presence of a single product (Supplementary Fig. S2a,b). The amplified PCR products showed single bands when run on 1.5% agarose gel (Supplementary Fig. S3). The sequences of the amplified PCR products confirmed the specificity of the primers.

**Table 2:** Primer efficiency for housekeeping genes in *Celmisia* and *Chionochloa*.

Gene	Primer sequences	Length	Tm	E (amp)	Efficiency (%)	R2
***Celmisia***						
*EF*	5′ CCGCCACTTCCATCTCTACAATCTA-3′ 5′ AGCAACACACTCATACCACTGACT-3′	244	60	1.995	99.5	0.9999
*eIF*	5′-TCCTACTTATGTTGTTGGTGTCAATGC 5′-CAGTGTAAGAGTGAGTGGTGGTCAT	164	60	2.087	108.7	0.9995
*GAPDH*	5′-ATACTTTGTCGTCATCGTCATCTTCAC-3′ 5′-CCTGGTCGGTGGATATTGTTGTAGA-3′	202	58	2.055	105.5	0.99983
*U18S*	5′-GCTGAAACTTAAAGGAATTGACGGAAG-3′ 5′-TTGAAGACCAACAATTGCAATGATCTATC-3′	320	60	1.954	95.4	0.9998
*SAND*	5′-GACATGACACCATTGCTTGG-3′ 5′-GAGTCAGCAACATCCTGCAA-3′	161	58	1.972	97.2	0.99998
*MTP*	5′-GCCAAGGGAAATAGATGCAA-3′ 5′-TCAAGCACCAGATCAGCATC-3′	162	58	1.977	97.7	0.9999
*PGK1*	5′-GCTTCCGCTTCCTGTACTCCAA-3′ 5′-GGCTTCCTGTCTACCACTTGATCT-3′	182	60	1.99	99	0.99996
*PP2A*	5′-CCAACGCTGCATGGTTCCTCT-3′ 5′-GGTTCCAGTGAGCCTGAATGTTC-3′	191	60	2.116	110.6	0.9964
***Chionochloa***						
*EF*	5′-CATGCTCTCCTTGCTTTCACTCTT-3′ 5′-CTTGTACCAGTCAAGGTTGGTGGAC-3′	228	60	2.077	107.7	0.9992
*GAPDH*	5′-CTTCCTGCCCTTAATGGAAAGTTG-3′ 5′-GTCACCCTGGAAGTCAGTGGAAAC-3′	210	58	2.073	107.3	0.9991
*U18S*	5′-GCTGAAACTTAAAGGAATTGACGGAAG-3′ 5′-TTGAAGACCAACAATTGCAATGATCTATC-3′	320	60	1.965	96.5	0.9999
*NBP*	5′-GGTTAGTCAAGAAGAGGCATACAAGTC-3′ 5′-ACAGCAGCAGCAGATGGAGTG-3′	198	60	1.984	98.4	0.99988
*ExP*	5′-GCACCACCTCTGAAGCCAAG-3′ 5′-CCACATATAAGACAACCAGTCATCG-3′	213	58	1.968	96.8	0.99998
*THP*	5′-AGAAGGAACTTGGTGGCAGACTC-3′ 5′-TCCGTGAAGGTGGTTGACATTGT-3′	176	60	2.045	104.5	0.99998
*Actin*	5′-TGAGCATGGAATTGTGAGCAACTG-3′ 5′-TGGATGGCAACATACATAGCAGGA-3′	198	60	2.003	100.3	0.99997
*B*-*Tubulin*	5′-GAGTGGAGTCACATGCTGCCTAA-3′ 5′-GACCTCCTTCGTGCTCATCTTCC-3′	100	60	1.974	97.4	0.9998

### Expression analysis of the candidate reference genes

**Figure 2:**
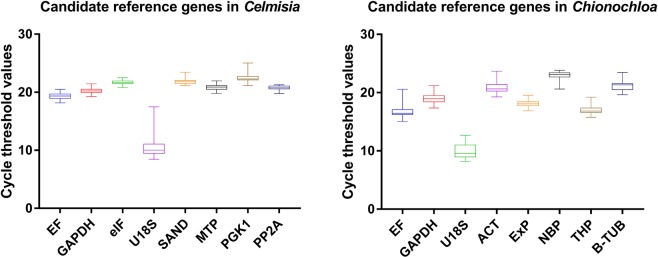
Cycle threshold values for candidate reference genes in *Celmisia* and *Chionochloa* are shown in box and whisker plots. The bars represent maximum and minimum C_q_ value.

The raw quantification cycle (C_q)_ values for each of the eight candidate reference genes in *Celmisia* and *Chionochloa* are shown in Fig. 2 (Supplementary Data S4). In *Celmisia*, the C_q_ values varied from 8.44 to 25.10 while the mean C_q_ value ranged from 10.63 to 22.44. *18S* showed the greatest variance amongst the eight candidate reference genes. In *Chionochloa*, the C_q_ values ranged from 8.15 to 23.15 with an average C_q_ value ranging from 10.02 to 22.87. In *Chionochloa*, *18S* had the highest variability amongst the eight candidate reference genes. The stability was analysed in comparison with developmental stage (vegetative and flowering), time-course (January, May, September, and March), and attitudinal changes (1520, 1350, 1070 m at Mt Hutt and near sea level at the University of Canterbury).

### Stability of the candidate reference genes in *Celmisia*

**Figure 3:**
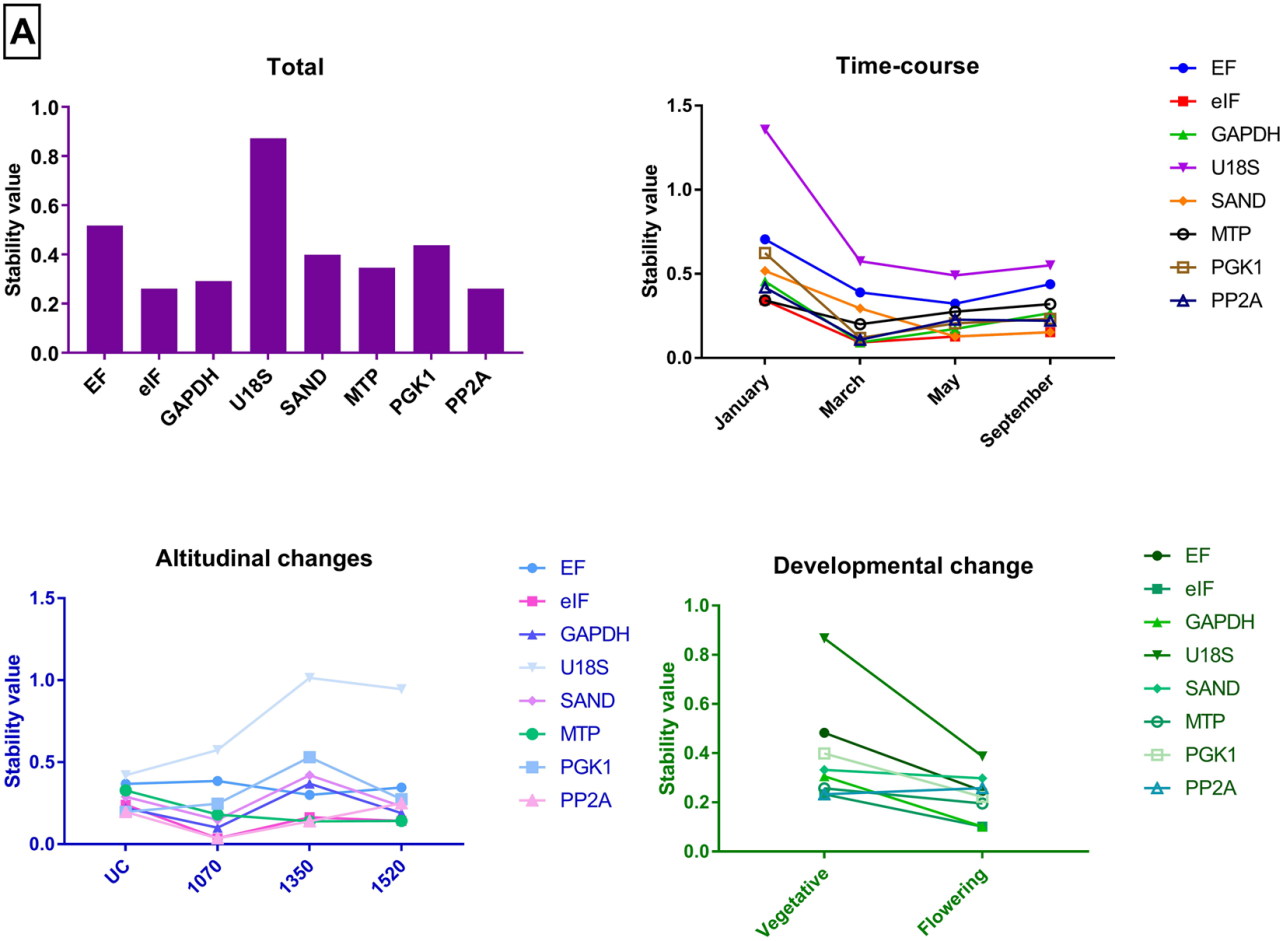
GeNorm analysis of the candidate reference genes in 48 collected *Celmisia* samples across different experimental groups (Total, time-course, altitude and developmental stage). The gene with the lowest stability value is the most stable gene.

**Figure 4:**
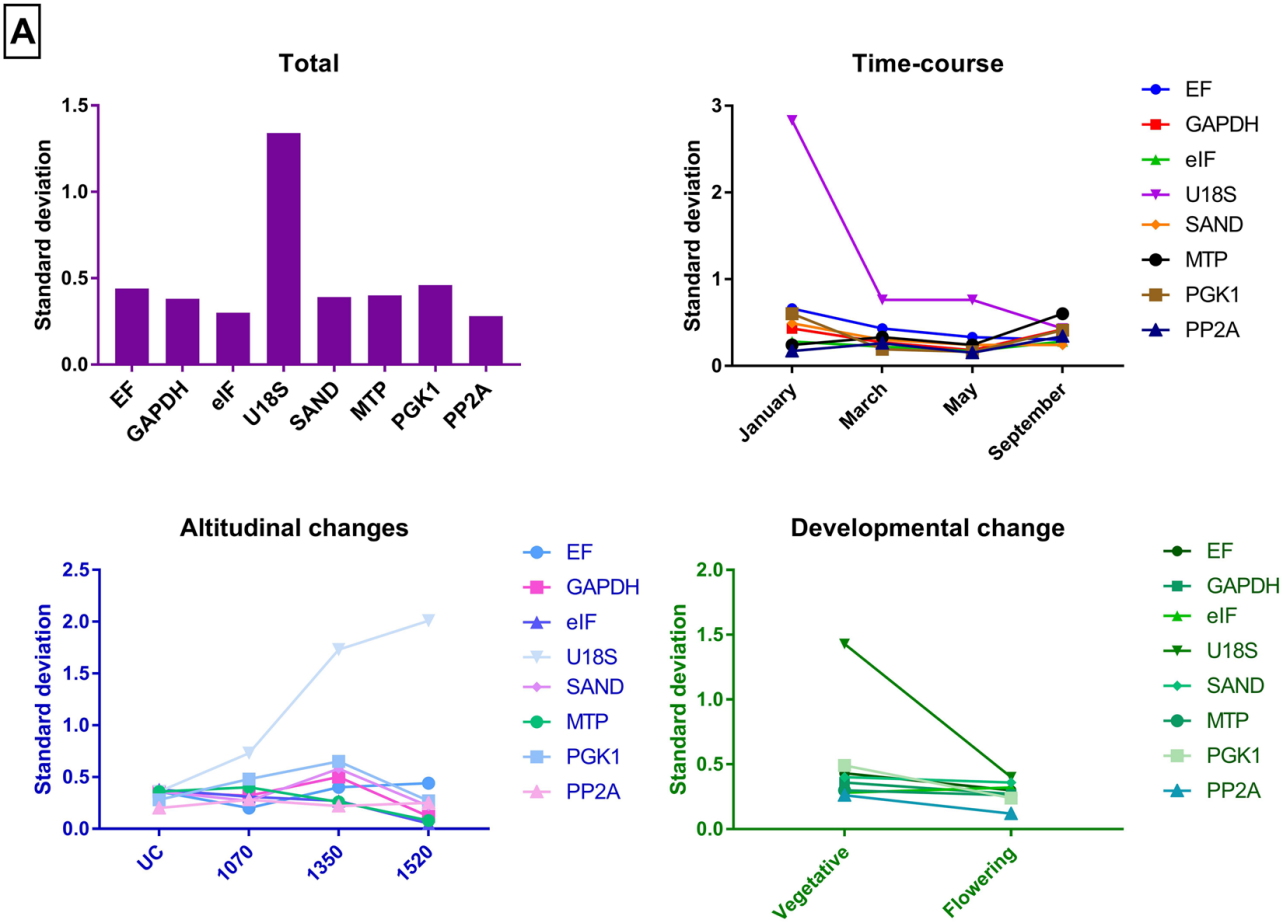
BestKeeper analysis of the candidate reference genes in 48 *Celmisia* samples tested across different experimental groups (Total, time-course, altitude and developmental stage). The gene with the lowest standard deviation value is the most stable gene.

A potential reference gene should have a stable expression across the developmental, altitudinal and seasonal changes. GeNorm analysis showed that *eIF* and *PP2a* were the more stable genes across all the different comparisons (Fig. 3). The optimal number of reference gene(s) required for normalisation in all the experimental manipulations was calculated using geNorm. The calculated pairwise variation for all the different experimental groups indicated two genes were sufficient for normalisation as the cut-off value was below 0.15 even when additional reference genes were added (V2/3 = 0.000 and V3/4 = 0.000). NormFinder analysis showed *SAND* and *PP2a* as the higher stability genes compared to other genes in the collected samples (Table 3), whereas Bestkeeper analysis pointed to *PP2a* as the most stable gene (Fig. 4). A similar ranking was seen when all the reference genes were analysed by the ΔC_t_ method (Table 4).

**Table 3:** NormFinder analysis for selection of candidate reference gene(s) in *Celmisia* and *Chionochloa*.

Ranking	Total		1520		1350		1070		UC		January		March		May		September		Vegetative		Flowering	
	Gene	M-value	Gene	M-value	Gene	M-value	Gene	M-value	Gene	M-value	Gene	M-value	Gene	M-value	Gene	M-value	Gene	M-value	Gene	M-value	Gene	M-value
***Celmisia***																						
1	*GAPDH*	0.101	*PGK1*	0.053	*MTP*	0.101	*eIF*	0.038	*U18S*	0.068	*PGK1*	0.100	*EF*	0.012	*eIF*	0.057	*SAND*	0.050	*GAPDH*	0.089	*eIF*	0.038
2	*SAND*	0.102	*SAND*	0.053	*GAPDH*	0.101	*MTP*	0.046	*MTP*	0.109	*EF*	0.124	*SAND*	0.032	*PP2A*	0.063	*GAPDH*	0.053	*PP2A*	0.089	*MTP*	0.092
3	*PP2A*	0.163	*MTP*	0.073	*PP2A*	0.191	*PGK1*	0.053	*SAND*	0.128	*SAND*	0.139	*PP2A*	0.039	*SAND*	0.066	*U18S*	0.122	*PGK1*	0.146	*PP2A*	0.118
4	*eIF*	0.237	*U18S*	0.077	*EF*	0.265	*SAND*	0.081	*PP2A*	0.158	*GAPDH*	0.145	*U18S*	0.079	*PGK1*	0.169	*MTP*	0.158	*U18S*	0.261	*SAND*	0.127
5	*PGK1*	0.330	*GAPDH*	0.272	*PGK1*	0.295	*GAPDH*	0.180	*PGK1*	0.242	*MTP*	0.465	*eIF*	0.295	*GAPDH*	0.187	*PP2A*	0.243	*EF*	0.311	*GAPDH*	0.141
6	*EF*	0.372	*eIF*	0.343	*eIF*	0.348	*EF*	0.322	*GAPDH*	0.259	*eIF*	0.599	*MTP*	0.325	*EF*	0.218	*EF*	0.353	*eIF*	0.360	*PGK1*	0.144
7	*MTP*	0.400	*PP2A*	0.372	*U18S*	0.561	*U18S*	0.474	*EF*	0.263	*PP2A*	0.651	*PGK1*	0.366	*MTP*	0.316	*eIF*	0.441	*SAND*	0.380	*EF*	0.294
8	*U18S*	1.319	*EF*	1.901	*SAND*	1.692	*PP2A*	0.757	*eIF*	0.355	*U18S*	2.281	*GAPDH*	0.753	*U18S*	0.673	*PGK1*	0.568	*MTP*	1.378	*U18S*	0.433
***Chionochloa***																						
**Ranking**	**Total**		**1520**		**1070**		**UC**		**January**		**February**		**March**		**May**		**September**		**Vegetative**		**Flowering**	
	**Gene**	**M-value**	**Gene**	**M-value**	**Gene**	**M-value**	**Gene**	**M-value**	**Gene**	**M-value**	**Gene**	**M-value**	**Gene**	**M-value**	**Gene**	**M-value**	**Gene**	**M-value**	**Gene**	**M-value**	**Gene**	**M-value**
1	*TUB*	0.309	*TUB*	0.208	*THP*	0.078	*TUB*	0.201	*NBP*	0.037	*ExP*	0.050	*ExP*	0.096	*ExP*	0.041	*ExP*	0.206	*THP*	0.194	*THP*	0.296
2	*ExP*	0.325	*THP*	0.210	*ACT*	0.128	*NBP*	0.239	*THP*	0.068	*EF*	0.143	*THP*	0.197	*TUB*	0.041	*U18S*	0.303	*TUB*	0.209	*ExP*	0.363
3	*THP*	0.365	*GAPDH*	0.211	*EF*	0.168	*U18S*	0.251	*EF*	0.152	*U18S*	0.143	*EF*	0.262	*THP*	0.291	*NBP*	0.304	*ACT*	0.353	*TUB*	0.372
4	*EF*	0.370	*ACT*	0.264	*NBP*	0.326	*GAPDH*	0.278	*ExP*	0.167	*NBP*	0.161	*GAPDH*	0.410	*GAPDH*	0.307	*EF*	0.386	*U18S*	0.411	*NBP*	0.387
5	*GAPDH*	0.384	*NBP*	0.299	*GAPDH*	0.480	*ACT*	0.401	*U18S*	0.288	*GAPDH*	0.183	*NBP*	0.412	*NBP*	0.351	*TUB*	0.458	*GAPDH*	0.432	*GAPDH*	0.411
6	*NBP*	0.432	*U18S*	0.364	*ExP*	0.514	*ExP*	0.482	*ACT*	0.585	*ACT*	0.195	*ACT*	0.435	*ACT*	0.439	*GAPDH*	0.462	*EF*	0.433	*EF*	0.449
7	*ACT*	0.479	*ExP*	0.588	*TUB*	0.788	*EF*	0.484	*TUB*	0.820	*THP*	0.431	*U18S*	0.496	*U18S*	0.446	*ACT*	0.543	*ExP*	0.437	*U18S*	0.534
8	*U18S*	0.887	*EF*	0.734	*U18S*	1.064	*THP*	0.726	*GAPDH*	1.539	*TUB*	0.495	*TUB*	0.528	*EF*	0.805	*THP*	0.589	*NBP*	0.833	*ACT*	0.966

**Table 4:** ΔCt analysis for selection of candidate reference gene(s) in *Celmisia* and *Chionochloa*.

Ranking	Total		1520		1350		1070		UC		January		March		May		September		Vegetative		Flowering	
	Gene	Std dev	Gene	Std dev	Gene	Std dev	Gene	Std dev	Gene	Std dev	Gene	Std dev	Gene	Std dev	Gene	Std dev	Gene	Std dev	Gene	Std dev	Gene	Std dev
***Celmisia***																						
1	*GAPDH*	0.63	*SAND*	0.61	*PP2A*	0.71	*PP2A*	0.4	*PP2A*	0.32	*GAPDH*	0.95	*eIF*	0.39	*GAPDH*	0.36	*SAND*	0.4	*GAPDH*	0.62	*MTP*	0.29
2	*PP2A*	0.64	*PP2A*	0.63	*GAPDH*	0.73	*SAND*	0.4	*PGK1*	0.34	*PP2A*	0.96	*PGK1*	0.4	*eIF*	0.36	*PP2A*	0.41	*PP2A*	0.62	*GAPDH*	0.33
3	*eIF*	0.66	*GAPDH*	0.66	*MTP*	0.74	*eIF*	0.4	*GAPDH*	0.35	*SAND*	1	*PP2A*	0.4	*SAND*	0.38	*eIF*	0.42	*SAND*	0.64	*PGK1*	0.33
4	*SAND*	0.69	*PGK1*	0.66	*eIF*	0.74	*GAPDH*	0.42	*eIF*	0.38	*eIF*	1.03	*GAPDH*	0.4	*PGK1*	0.4	*PGK1*	0.43	*eIF*	0.65	*PP2A*	0.33
5	*PGK1*	0.77	*MTP*	0.71	*SAND*	0.8	*MTP*	0.45	*SAND*	0.45	*MTP*	1.15	*MTP*	0.57	*PP2A*	0.41	*GAPDH*	0.5	*MTP*	0.7	*eIF*	0.35
6	*MTP*	0.78	*eIF*	0.74	*EF*	0.87	*PGK1*	0.59	*MTP*	0.46	*EF*	1.18	*SAND*	0.61	*EF*	0.5	*MTP*	0.6	*PGK1*	0.8	*EF*	0.35
7	*EF*	0.87	*EF*	0.81	*PGK1*	1.07	*EF*	0.8	*EF*	0.48	*PGK1*	1.27	*EF*	0.69	*MTP*	0.53	*EF*	0.74	*EF*	0.87	*SAND*	0.47
8	*U18S*	1.94	*U18S*	2.75	*U18S*	2.47	*U18S*	1.14	*U18S*	0.57	*U18S*	3.32	*U18S*	1.13	*U18S*	0.99	*U18S*	0.88	*U18S*	2.02	*U18S*	0.65
***Chionochloa***																						
**Ranking**	**Total**		**1520**		**1070**		**UC**		**January**		**February**		**March**		**May**		**September**		**Vegetative**		**Flowering**	
	**Gene**	**Std dev**	**Gene**	**Std dev**	**Gene**	**Std dev**	**Gene**	**Std dev**	**Gene**	**Std dev**	**Gene**	**Std dev**	**Gene**	**Std dev**	**Gene**	**Std dev**	**Gene**	**Std dev**	**Gene**	**Std dev**	**Gene**	**Std dev**
1	*ExP*	0.8	*GAPDH*	0.78	*TUB*	0.75	*EF*	0.66	*GAPDH*	0.78	*ACT*	0.36	*EF*	0.53	*TUB*	0.57	*EF*	0.64	*TUB*	0.71	*EF*	0.81
2	*TUB*	0.83	*TUB*	0.84	*ExP*	0.8	*NBP*	0.69	*TUB*	0.84	*ExP*	0.39	*THP*	0.59	*EF*	0.57	*ExP*	0.68	*ExP*	0.72	*THP*	0.86
3	*THP*	0.83	*ExP*	0.86	*THP*	0.82	*TUB*	0.7	*ExP*	0.86	*U18S*	0.4	*GAPDH*	0.63	*GAPDH*	0.65	*THP*	0.7	*THP*	0.79	*ExP*	0.87
4	*EF*	0.83	*THP*	0.89	*GAPDH*	0.86	*ExP*	0.7	*THP*	0.89	*EF*	0.42	*ExP*	0.73	*ExP*	0.68	*TUB*	0.81	*EF*	0.85	*GAPDH*	0.89
5	*GAPDH*	0.87	*NBP*	1	*ACT*	0.94	*THP*	0.8	*NBP*	1	*NBP*	0.42	*ACT*	0.78	*ACT*	0.72	*ACT*	0.86	*ACT*	0.86	*TUB*	0.93
6	*NBP*	0.92	*ACT*	1.06	*NBP*	1.02	*ACT*	0.88	*ACT*	1.06	*TUB*	0.47	*NBP*	0.81	*THP*	0.76	*GAPDH*	0.88	*GAPDH*	0.86	*NBP*	0.98
7	*ACT*	0.93	*EF*	1.29	*EF*	1.22	*GAPDH*	0.9	*EF*	1.29	*GAPDH*	0.67	*U18S*	0.86	*NBP*	0.83	*NBP*	0.94	*NBP*	0.88	*ACT*	1.01
8	*U18S*	1.39	*U18S*	2.26	*U18S*	1.6	*U18S*	1.16	*U18S*	2.26	*THP*	0.76	*TUB*	0.9	*U18S*	1.22	*U18S*	1.02	*U18S*	1.3	*U18S*	1.5

RefFinder generated a comprehensive list of ranking order of the candidate reference genes. In the comparative analysis for leaves from vegetative and flowering samples, *PP2a* and *GAPDH* were found to be the two most stable reference genes. Under altitudinal translocations, *PP2a*, *eIF* and *GAPDH* were the most stable candidate reference genes. However, the time-course analysis of the candidate reference genes showed *eIF*, *SAND* and *GAPDH* as the better reference genes for data normalisation. From the analysed data, *PP2a* and *GAPDH* were found to be best for gene expression data normalisation in *Celmisia* (Table 5).

**Table 5:** Comprehensive ranking of candidate reference genes in *Celmisia* and *Chionochloa*.

Ranking	Total		1520		1350		1070		UC		January		March		May		September		Vegetative		Flowering	
	Gene	M-value	Gene	M-value	Gene	M-value	Gene	M-value	Gene	M-value	Gene	M-value	Gene	M-value	Gene	M-value	Gene	M-value	Gene	M-value	Gene	M-value
***Celmisia***																						
1	*PP2A*	1.57	*SAND*	2	*PP2A*	1.32	*PP2A*	1.32	*PP2A*	1	*PP2A*	1.86	*eIF*	1.41	*SAND*	1.86	*eIF*	1.19	*PP2A*	1.565	*MTP*	1.73
2	*GAPDH*	1.73	*eIF*	2.55	*MTP*	2.21	*eIF*	2.63	*PGK1*	1.68	*GAPDH*	2.63	*SAND*	2	*eIF*	2.06	*GAPDH*	1.57	*GAPDH*	2.378	*GAPDH*	2.21
3	*eIF*	2.21	*MTP*	2.78	*GAPDH*	3.16	*SAND*	2.63	*GAPDH*	3.57	*MTP*	2.78	*GAPDH*	2.21	*PP2A*	2.45	*PGK1*	2.91	*eIF*	2.378	*PP2A*	2.63
4	*SAND*	3.56	*PP2A*	3.16	*SAND*	3.66	*GAPDH*	3.31	*SAND*	4.4	*eIF*	2.78	*PP2A*	3.22	*PGK1*	3.34	*PP2A*	4.23	*SAND*	2.943	*PGK1*	3.13
5	*MTP*	5.38	*GAPDH*	3.41	*eIF*	3.66	*EF*	4.3	*eIF*	4.76	*EF*	4.14	*PGK1*	5.23	*GAPDH*	3.36	*MTP*	5.23	*MTP*	3.873	*eIF*	3.66
6	*PGK1*	5.69	*PGK1*	4.9	*EF*	4.9	*MTP*	5.23	*MTP*	5.96	*SAND*	4.16	*MTP*	5.73	*EF*	6.19	*SAND*	5.66	*PGK1*	6.236	*EF*	5.23
7	*EF*	6.48	*EF*	5.66	*PGK1*	7	*PGK1*	6.24	*U18S*	6.73	*PGK1*	6.48	*EF*	7	*MTP*	6.48	*EF*	6.45	*EF*	6.735	*SAND*	7
8	*U18S*	8	*U18S*	8	*U18S*	8	*U18S*	8	*EF*	6.74	*U18S*	8	*U18S*	8	*U18S*	8	*U18S*	7.74	*U18S*	8	*U18S*	8
***Chionochloa***																						
**Ranking**	**Total**		**1520**		**1070**		**UC**		**January**		**February**		**March**		**May**		**September**		**Vegetative**		**Flowering**	
	**Gene**	**M-value**	**Gene**	**M-value**	**Gene**	**M-value**	**Gene**	**M-value**	**Gene**	**M-value**	**Gene**	**M-value**	**Gene**	**M-value**	**Gene**	**M-value**	**Gene**	**M-value**	**Gene**	**M-value**	**Gene**	**M-value**
1	*ExP*	1.19	*THP*	1.5	*TUB*	1.57	*EF*	0.201	*GAPDH*	1.57	*ACT*	1.73	*EF*	1.19	*GAPDH*	2.28	*EF*	1	*ExP*	1.41	*THP*	1.68
2	*THP*	2.06	*ExP*	2.45	*ExP*	2.63	*GAPDH*	0.482	*ExP*	2.06	*ExP*	1.86	*THP*	1.41	*ExP*	2.51	*ExP*	2.21	*THP*	2.06	*ExP*	1.97
3	*TUB*	2.78	*EF*	2.91	*GAPDH*	2.99	*U18S*	0.726	*TUB*	2.34	*NBP*	2.34	*GAPDH*	3.41	*TUB*	2.63	*THP*	3.22	*TUB*	2.24	*EF*	1.97
4	*EF*	3.72	*ACT*	3.98	*THP*	3.22	*ACT*	0.484	*NBP*	3.34	*U18S*	3.13	*ExP*	3.72	*EF*	2.66	*TUB*	3.72	*ACT*	4.36	*GAPDH*	3.46
5	*NBP*	5.24	*U18S*	4.76	*NBP*	3.83	*ExP*	0.278	*THP*	4	*EF*	4.47	*ACT*	5.23	*THP*	3.35	*ACT*	4.95	*EF*	5.09	*TUB*	5.38
6	*ACT*	5.6	*GAPDH*	4.76	*ACT*	5.44	*NBP*	0.239	*ACT*	6	*TUB*	5.73	*NBP*	5.42	*ACT*	4.68	*GAPDH*	5.89	*NBP*	5.21	*NBP*	5.63
7	*GAPDH*	5.69	*TUB*	5.05	*U18S*	5.66	*THP*	0.401	*EF*	7.24	*GAPDH*	7.24	*U18S*	7.24	*NBP*	6.74	*U18S*	6.26	*GAPDH*	5.83	*ACT*	6.19
8	*U18S*	8	*NBP*	7	*EF*	7.24	*TUB*	0.251	*U18S*	7.74	*THP*	7.74	*TUB*	7.74	*U18S*	8	*NBP*	7	*U18S*	7.74	*U18S*	8

### Stability of reference genes in *Chionochloa*

**Figure 5:**
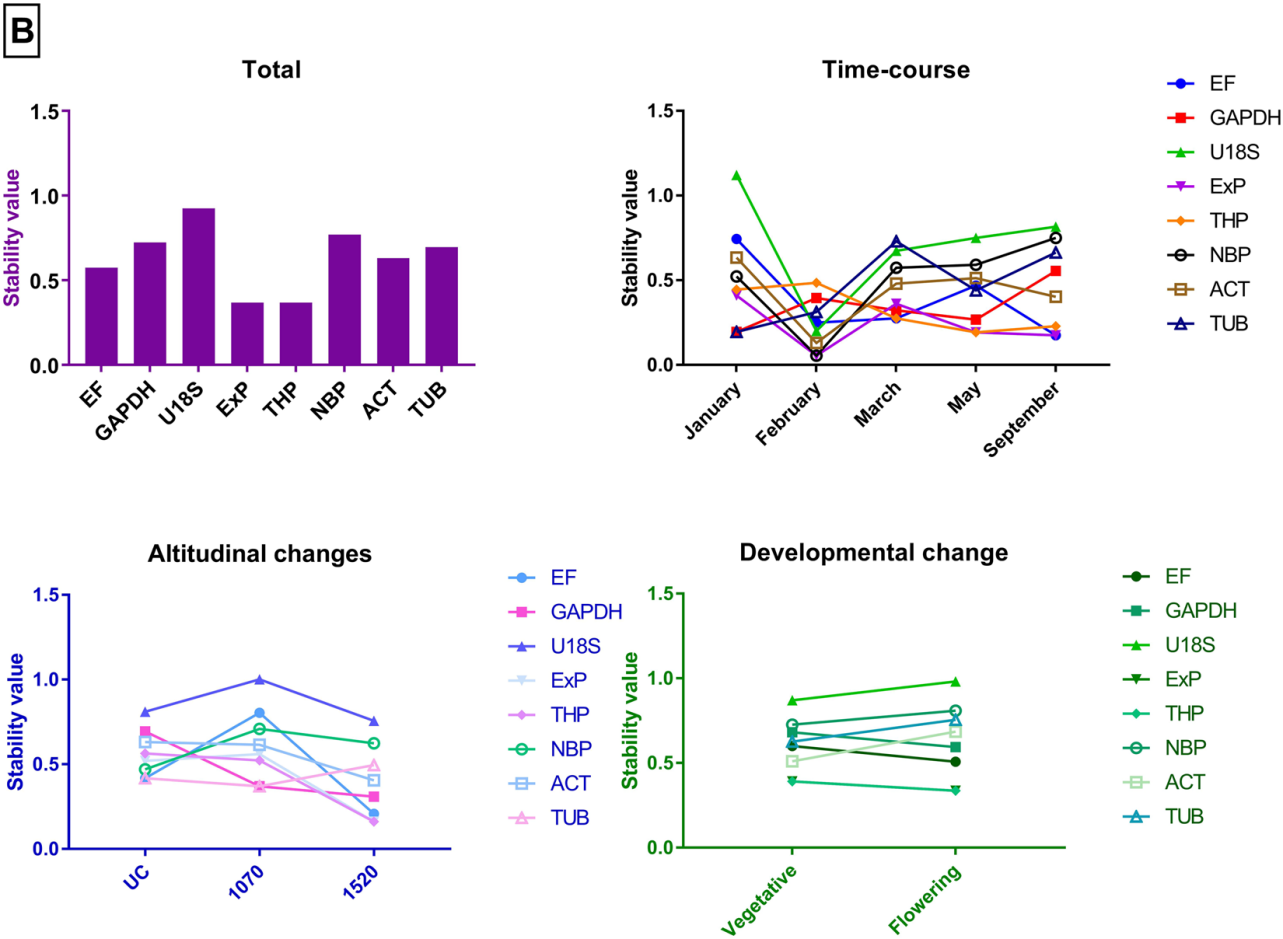
GeNorm analysis of the candidate reference genes in 54 collected *Chionochloa* samples across different experimental groups (Total, time-course, altitude and developmental stage). The gene with the lowest stability value is the most stable gene.

**Figure 6:**
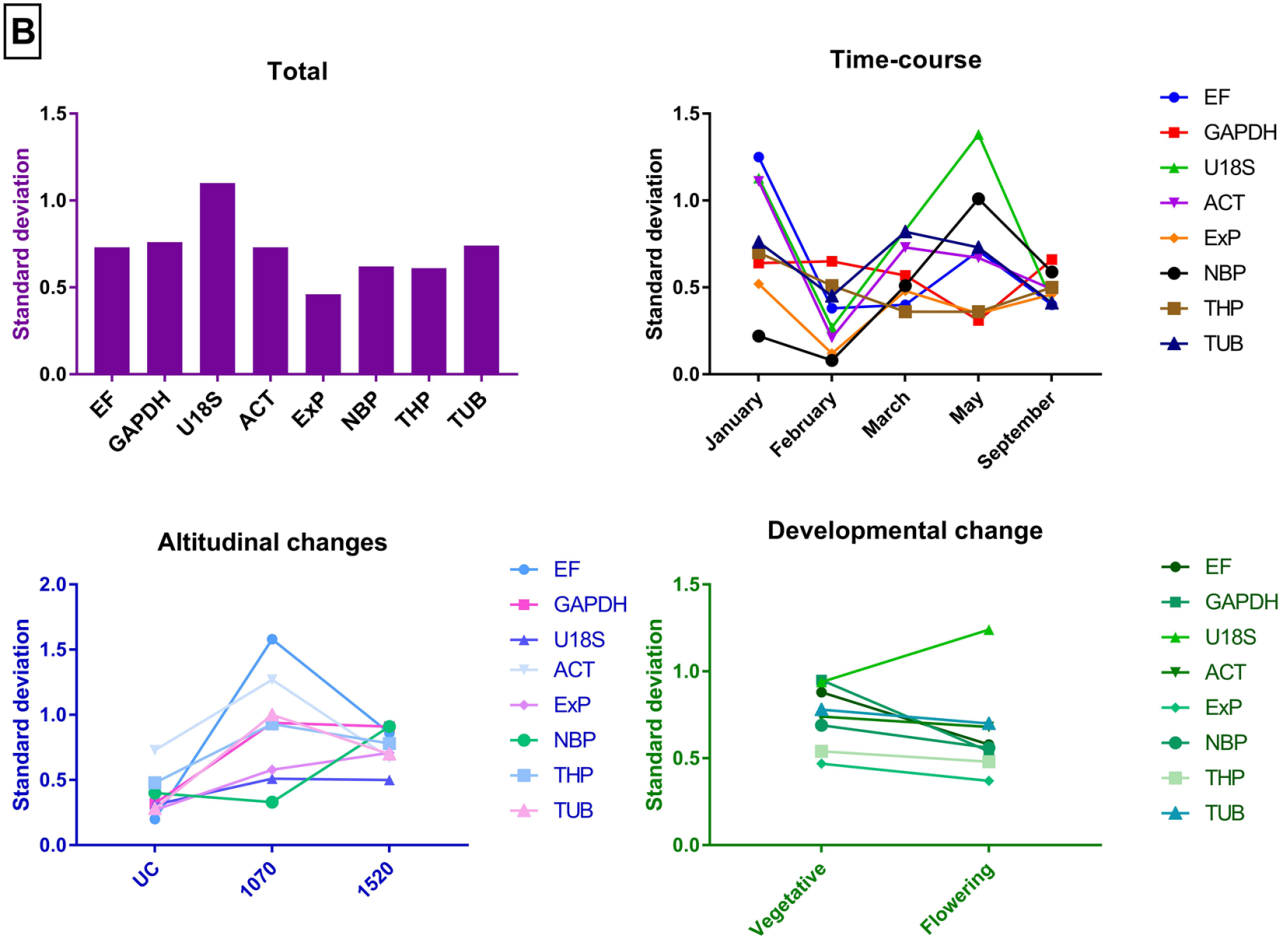
BestKeeper analysis of the candidate reference genes in 54 *Chionochloa* samples tested across different experimental groups (Total, time-course, altitude and developmental stage). The gene with the lowest standard deviation value is the most stable gene.

The geNorm analysis showed *ExP* and *THP* to be the most stable reference genes in most of the experimental groups (Fig. 5). *TUB* and *GAPDH* were found to be more stable in the January samples collected from the University of Canterbury and 1070 m base of Mt Hutt, while *EF* was also found to be an equally stable reference gene in the samples collected in March and September. Similar to *Celmisia*, the pairwise variation for an optimal number of reference genes was found to be less than 0.15 for adding additional genes more than two. This suggests that two reference genes are sufficient to normalise the gene expression data from *Chionochloa* samples. NormFinder identified *EF* as the most stable gene across all the samples followed by *TUB* except for samples collected from 1070 and January time point. *ExP* and *ACT* were more stable in the January and February samples, respectively (Table 3). According to the Best Keeper analysis based on standard deviation (std dev) values, *ExP* was the most stable gene for all the samples (Fig. 6). The coefficient of variation was lowest for *NBP* in most of the samples across different experimental groups. However, the ranking of NBP was different in vegetative and flowering samples. Interestingly, *U18S* was ranked highest for samples collected from 1520 m base of Mt Hutt.

During the time course experimental group, *THP*, *EF*, and *GAPDH* were found to be more stable in the March, September, and May samples. The ranking for the candidate reference genes using the ∆C_t_ method was variable in different experimental groups as shown in Table 4. *EF* was consistently a stable gene in most of the experimental groups. Similar variability can be seen in the comprehensive ranking order generated by RefFinder across all the different experimental groups. *ExP* and *THP*, or either of them were always present in the top two positions in terms of expression stability. Based on the relative fold change in the expression levels and outputs from the statistical analysis, *ExP* and *THP* were selected as the best gene combination for normalisation of the gene expression analysis (Table 5).

### Validation of the selected reference gene

**Figure 7:**
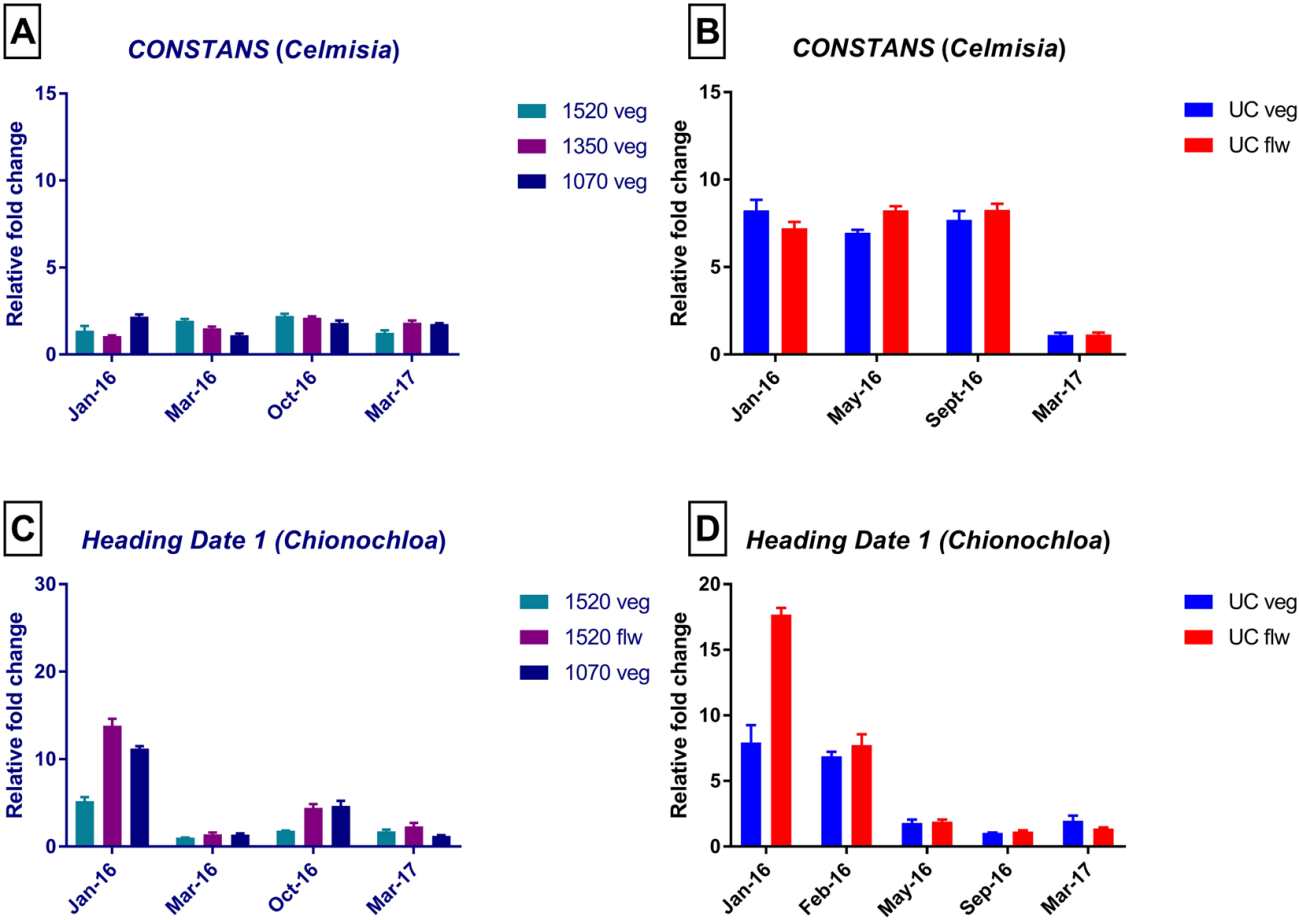
Validation of the selected reference genes in *Celmisia* and *Chionochloa*. (**A**) Expression analysis of *CO* in samples collected from Mt Hutt using *PP2a* and *GAPDH*. (**B**) Expression analysis of *CO* in vegetative and flowering samples collected from the University of Canterbury using *PP2a* and *GAPDH*. (**C**) Expression analysis of *Hd1* in vegetative and flowering samples collected from Mt Hutt using *ExP* and *THP*. (**D**) Expression analysis of *Hd1* in vegetative and flowering samples collected from the University of Canterbury using *ExP* and *THP*. The data represent the mean ± S.D. of two independent biological replicates, each with three technical replicates. Legends ending with flw or veg corresponds to the fate of the leaves used in the analysis as flowering or vegetative respectively.

**Figure 8:**
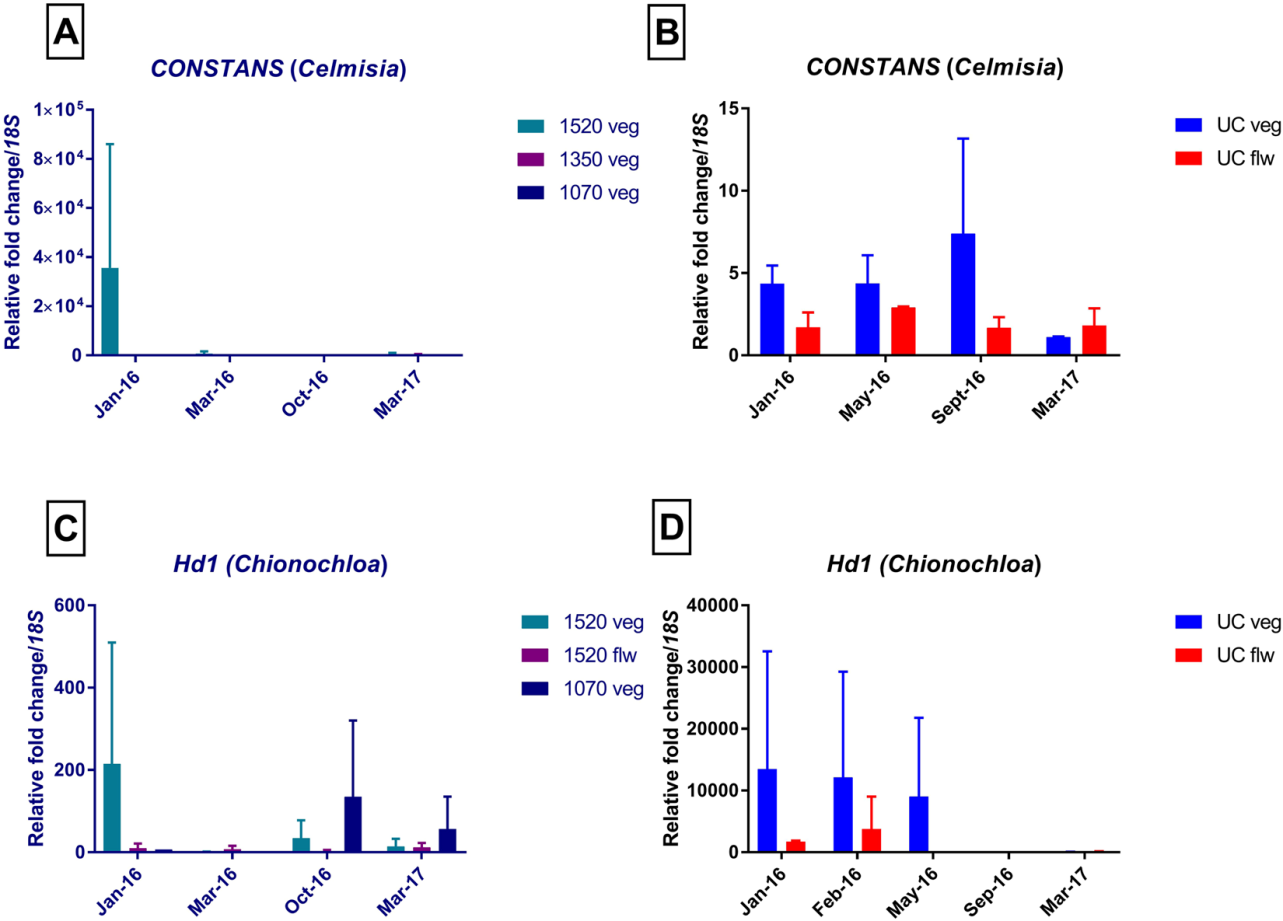
Expression analysis of *CO* and *Hd1* in *Celmisia* and *Chionochloa* using the least stable reference gene, *18S ribosomal gene*. (**A**) *CO* expression in samples collected from Mt Hutt. (**B**) *CO* expression in vegetative and flowering samples collected from the University of Canterbury. (**C**) *Hd1* expression in vegetative and flowering samples collected from Mt Hutt. (**D**) *Hd1* expression in vegetative and flowering samples collected from the University of Canterbury. The data represent the mean ± S.D. of two independent biological replicates, each with three technical replicates. Legends ending with flw or veg corresponds to the fate of the leaves used in the analysis as flowering or vegetative respectively.

The reliability of the selected reference genes was further verified by analysing the expression profile of *CONSTANS* (*CO*) and *Heading date 1* (*Hd1*). *Heading date 1* is an orthologue of *CO* present in monocots^66^. Relative expression levels of *CO* and *Hd1* were normalised using the normalisation factor derived from the two most stable reference genes (*PP2a* and *GAPDH* in *Celmisia*, and *ExP* and *THP* in *Chionochloa*) (Fig. 7), and the least stable-reference gene (*U18S*) (Fig. 8).

The expression pattern of *CO* in *Celmisia* was generally found to be similar to its ortholog of *Hd1* in *Chionochloa* when normalised with selected reference genes in the respective species (Fig. 7). The expression patterns of *CO* and *Hd1* varied across the season (Fig. 7B,D) (P-value < 0.0001). The expression of *Hd1* was significantly greater in the samples collected in January compared to other time points from the plants at the University of Canterbury (UC) and Mt Hutt (P-value < 0.0001) (Fig. 7C and D). Irrespective of whether the plants subsequently flowered or not, the patterns for *CO* and *Hd1* expression were the same in the leaves collected over time from UC in *Celmisia* (Fig. 7B) (P-value < 0.0001, F = 349.6). Expression of *Hd1* was much greater in leaves from the *Chionochloa* plants that flowered in the next season compared to leaves from plants that remained vegetative.

The expression data was also normalised using *U18S*, the least stable candidate gene in *Chionochloa* and *Celmisia*. Because the expression of *U18S* was itself highly variable, this is reflected in the variable expression of *CO* and *Hd1* in both the species in all the experimental groups as seen in the relative fold changes when comparing the y-axes in Figs 7 and 8. There were no significant differences in the expression of *CO* or *Hd1* at different time points in either species. Consequently, normalising the data using *U18S* would lead to inaccurate conclusions.

## Discussion

RT-qPCR is a widely used technique to study transcript abundance of a particular gene in distinct biological samples. Due to its high sensitivity and reliability, it is considered to be the gold standard for detecting and quantifying the expression pattern of gene(s) of interest. The choice of an appropriate normalisation factor is a key step in the RT-qPCR analysis to obtain accurate interpretation of the results. The normalisation factor is based on reference gene(s). Reference gene(s) are selected based on the prerequisite that they will be stably expressed in all the tissues/cells, and unaffected by experimental manipulation. Thus, the majority of the traditionally used reference genes are metabolic genes involved with basal cellular activities such as carbon metabolism, cellular structure maintenance and protein translation. *Actin*, *elongation factor 1α*, *β*-*tubulin*, *glyceraldehyde*-*3*-*phosphate dehydrogenase* and *ubiquitin* have been repeatedly used for RT-qPCR analysis in plants, as they are expected to be uniformly expressed in different tissues and organs. Consequently, variability in the expression of a reference gene can demonstrate the variability introduced to samples due to imperfections of the technology and sample preparation steps.

There are several steps during an RT-qPCR analysis where it is possible to introduce variations in the final results including mRNA extractions, cDNA synthesis, the PCR procedure, and design of primers^67^. Most researchers would agree that these potential variations can be reduced through the use of appropriate reference genes. These reference genes, with proper validation, can be used to correct such methodological errors in an effective and convenient manner. This ensures that any variation is covered to a similar extent in controls and experimental treatments.

However, there is no single gene known that remains stable in diverse experimental conditions, and use of a single reference gene as the normalisation factor can lead to accumulation of relatively large errors in the interpretation of the gene expression data^47^. Unfortunately, an awareness of the importance of systematic validation of appropriate reference genes has still not completely permeated the scientific community. In one study in 2008, only 3.2% of 188 papers published in plant science journals from July 2007 to December 2007 used a validated reference gene^27^. Even today, many of the RT-qPCR analyses published in *Plant Physiology* and *The Plant Cell* in the past six months have used only a single reference gene, many of which were not reported to have been validated (Supplementary Fig. S5).

There are already reports of traditional reference genes that, while shown to be stably expressed under various biotic and abiotic stresses, have differential expression under other experimental conditions^35,43,47^. Studies using Arabidopsis have demonstrated variations in reference gene stability under different experimental conditions^20^. Similar studies have also been published in rice^30^, citrus^68^, pea^69^, ryegrass^70^, carrot^71^, soybean^45,46^ and maize^34^ suggesting variation in the ranking of the reference gene stability can be attributed to different stresses applied to the plants of interest. In one such study in citrus, different pairs of reference genes provided better normalisation factors under different biotic stresses^68^. Such studies show that the use of non-statistically validated reference genes can negatively impact the normalisation procedure and introduce errors in the expression patterns of the genes of interest. The most appropriate reference gene(s) should be properly identified and validated in all biological samples across the different experimental groups.

However, field conditions differ from controlled environment conditions, and this adds another level of complexity to the selection of appropriate reference genes. Systematic validation of the reference genes in real-time PCR analyses in non-model plant species in the field is required as pointed out in Tashiro *et al*.^42^. Tashiro *et al*., identified stably expressed genes in different grapevine cultivars emphasising the importance of validation of ranking stability of reference genes in a heterologous plant population. There are limited reports of selection of reference genes for normalisation in experimentally manipulated plants in the field^42,70,72^, and no such study has been published for masting plants.

We aimed to identify stably expressed genes to avoid the limitations imposed by (a) inappropriate selection, (b) improper validation, and (c) field conditions in order to study gene expression in the non-model masting plants under prevailing environmental conditions. Plants used in this study, *Chionochloa* and *Celmisia*, were translocated to different altitudes to provide conditions predicted to promote flowering^52^. It was interesting to note that only the *Celmisia* and *Chionochloa* plants translocated to sea level flowered heavily, along with a few of the *Chionochloa* plants translocated to the 1520 m site at Mt Hutt. Candidate reference genes were selected and analysed in different temporal-spatial manipulations for both of the species using four different statistical algorithms - geNorm, NormFinder, Bestkeeper, and ∆C_t_. Since the ranking of stably expressed genes can differ across these four algorithms due to varied strengths and application conditions of each of the software^55,58^, it is often difficult for researchers to identify the most stable reference genes. In cases where multiple algorithms are used, a comprehensive ranking is required for the selection of appropriate reference genes. RefFinder is a web-based analysis tool that generates a final comprehensive ranking of reference genes based on their stability output as evaluated by the four independent algorithms. The global ranking is based on the integration of the geometric mean (GM) of the ranking values obtained from the four statistical tools. Importantly, RefFinder evaluates the ranking independent of the unrelated cut-offs and appropriate weights of the GM of the ranking values^28,73^.

The candidate reference genes were selected based on their previous validation in related species specifically in response to temperature shifts. Even then, the stability ranking of the candidate reference genes in *Celmisia* and *Chionochloa*, as determined by geNorm, NormFinder, BestKeeper and ∆C_t_, was found to be variable in different experimental groups. This variation may arise due to the different limitations of the statistical algorithms used in each program.

In *Chionochloa*, the geNorm analysis suggests that all the selected reference genes could be used as a potential normalisation factor as they have stability values of less than 1.5, an indicator of stably expressed reference genes. Reference genes showed a similar range of stability values during altitudinal and developmental shifts (Fig. 3). During the time-course analysis, an increase in the stability of reference genes was observed from January to February. Such change can be attributed to post-translocation effects. Since most of the selected reference genes regulate internal metabolism of the plants, the increase in the stability of the reference genes may then correspond to a plant’s ability to adapt to the new environment. Furthermore, either *ExP* or *THP* regularly showed up as the best normalisation factor for all the collected *Chionochloa* samples with other statistical tests as well. Two reference genes, *ExP* and *THP*, were found to be the most stably transcribed among all the samples, as previously reported for rice as well^63^ – both rice and *Chionochloa* are in the Poaceae. Since the pairwise variation for V3/4 was less than 0.15, *ExP* and *THP* were selected as the best candidate reference genes to be used as the normalisation factor for gene expression analysis in *Chionochloa*. *ExP* and *THP* code for an esterase and a mercury-dependent metal transporter, respectively. Esterases are generally associated with fatty acid degradation and modification. THP, a metal transporter protein, should have a stable expression under our experimental conditions. In our study, neither of these factors changed significantly in response to temperature changes.

In *Celmisia*, ranking of the candidate genes in all the experimental groups (Total) was found to be similar when analysed by the four algorithms. GeNorm identified *eIF* as the most stable gene in all the experimental samples. The stability ranking was found to be more variable in the NormFinder analysis. *PP2a* and *SAND* showed more stable expression across altitudinal and time-course change experiments. The BestKeeper and ΔC_t_ analysis identified *PP2A* and *GAPDH* as having the most stable expression pattern. Moreover, either *GAPDH* and/or *PP2a* always showed up in the top three positions in the comprehensive ranking, as the most stable reference genes across all the experimental groups. Based on the calculated pairwise variation output, *PP2a* and *GAPDH* were selected to validate the gene expression data of the flowering promoter *CONSTANS*. *PP2a* has already been established and validated to be one of the best candidate reference genes for normalisation in many plant species^74–76^. The protein is an essential component of cellular signalling pathways to achieve coordinated functioning between different cell types against oxidative stress. On the other hand, *GAPDH* is a classical reference gene used by many researchers, although its expression has been shown to vary under different environmental conditions^77^. Expression of *GAPDH* in coffee was found to be the most stable among all the tested candidates between different tissues^78^. In flax, *GAPDH* was found to be highly stable during different stages of plant development^36^. Expression of *GAPDH* may increase in conditions of abiotic and biotic stress where a plant requires more sugar utilisation and energy to stabilise its growth condition^79^. Since the sequence of *GAPDH* is highly conserved, it may be considered a good reference gene in many non-model plant species^80^. However, we highly recommend that *GAPDH* also be validated under experimental conditions in the field, as it was not the most stable gene in *Chionochloa*.

*CONSTANS*, was selected as a target gene to validate the credibility of the selected reference genes. *CONSTANS* is a CAAT-box transcription factor that has been shown to regulate photoperiodic control of flowering in Arabidopsis and in many other plant species^56^. The expression analysis of *CO* in *Celmisia* and *Chionochloa* was found to be similar to that in published reports when normalised using the two pairs of reference genes^81^. The two-way anova analysis suggests that the expression of *CO* was significantly different in all the seasons in distinct altitudes (P-value < 0.0001, F = 20.5). This is as expected as the transcription of the *CO* gene is regulated by photoperiodic signals. The expression was not significantly different between distinct altitudes of Mt Hutt. But the interaction between different seasons and altitude showed a positive response to the relative expression of *CO* in both species. The flowering genetic network is different between monocots and dicots even though the core genes remain the same^82^. This could explain the variation in the expression pattern of *Hd1* in the January leaf samples with different fates in *Chionochloa*, compared to *Celmisia* (Fig. 7D).

Even though the ranking order of the candidate reference genes may have differed, all the statistical programs consistently excluded *U18S*, ranking it as the most unstable gene among all the candidates. Expression analysis using the least stable gene (*U18S*) as the normalisation factor for both *Celmisia* and *Chionochloa*, showed significantly different results to the expression pattern using the selected reference gene pairs described above (comparing Figs 7 and 8). The expression values differed up to 14 log_2_ fold change in *Celmisia* and 12 log_2_ fold change in *Chionochloa* when analysed with *U18S* as the normalisation factor (Supplementary Fig. S7). These results clearly suggest that inappropriate selection of reference genes can introduce significant bias in the expression analysis and lead to misinterpretation of results. The use of *U18S* has also been discouraged among plant scientists due to its greater abundance in a sample compared to the target gene which can introduce greater errors in the analysis^26^.

## Conclusion: A Systematic Validation for Plants in Field Experiments

RT-qPCR is an extremely sensitive and important technique for real time amplification of transcripts. The selection of appropriate reference genes to use as the normalisation factor is equally as important as the technique. Since conditions in the field add a layer of complexity over those in controlled environments, it is crucial to verify the reliability of all potential reference genes in order to avoid misinterpretation of the genetic analysis. Discrepancies in the ranking order of candidate reference genes emphasises how crucial it is to validate every reference gene for any gene expression output in the natural environment. This work constitutes the first systematic analysis to identify, select and validate appropriate reference genes for genetic analysis in masting plants growing under prevailing environmental conditions. We have identified stably expressed reference genes from the draft transcriptomes of two masting plants, *Chionochloa* and *Celmisia*. These genes showed constant expression levels across seasonal, altitudinal and developmental changes and can be used to validate gene expression analysis. Our study will benefit both molecular and ecological research towards forecasting mast flowering in non-model plants under global climate change. Additionally, it will enable the New Zealand government to design efficient conservation measures aimed at the protection of endangered, endemic species.

## Methods

### Experimental design and plant material

Experiments were set up at the field site at Mt Hutt (43°32′S, 171°33′E). The control plots for *Celmisia* and *Chionochloa* were present at the 1350 m and 1070 m base of Mt Hutt, respectively. A set of 20 plants, randomly selected from the control plots, were moved to sea level (University of Canterbury, (43°31′S, 172°35′E). A similar set of *Chionochloa* was also translocated to an altitude of 1520 m on Mt Hutt, and a set of *Celmisia* plants was translocated to 1070 m and 1520 m. Leaf samples were collected four times throughout the year from each of the plants that were then segregated into two sets depending on whether the plants subsequently flowered (flowering sample) or remained vegetative (vegetative sample). Furthermore, an additional set of samples were also collected in the late summer for *Chionochloa* plants translocated to sea level to study the effect of a complete summer season on the flowering response. All of the samples were harvested and frozen using dry ice. The collected material was stored in −80 °C until subsequent analysis. Two independent biological replicates were used in the study, each consisting of a pool of three independent plants.

### Total RNA extraction

All the independent replicates were ground in mortar and pestle using liquid nitrogen. RNAzap was sprayed to clean the desk and types of equipment prior to use to inactivate the RNases present in the surroundings. Total RNA was extracted using the Trizol-chloroform method: 1 ml of Trizol along with 60 µl of 20% (w/v) Sarkosyl was added to the ground sample (~100 mg). The sample was centrifuged at 12000 g for 5 mins. The supernatant was transferred to a clean tube followed by addition of 200 µl of chloroform for phase separation. The mixture was allowed to stand for 3–4 mins followed by centrifugation at 12000 g for 15 mins. The aqueous phase from the above step was collected into a new tube and 500 µl of isopropanol was added to precipitate the RNA. The mixture was again centrifuged for 5 mins at 12000 g. The supernatant was discarded and the pellet was washed twice with 80% ethanol at 12000 rpm for 2 min. The clean, translucent pellet was air dried for 5–6 minutes. The RNA pellet was dissolved in RNase-free water in a dry bath at 65 °C and stored at −80 °C. The concentration and purity of the extracted RNA was assessed using a Nanodrop 2000c Spectrophotometer (Thermo Scientific, US). The absorbance ratios of A260/280 and A260/230 were the parameter used to check the quality of the extracted RNA. The values are required to be in between 2.0–2.1 for A260/280 and 2.0–2.2 for A260/230 for a pure RNA sample. The integrity of RNA was assessed using 1.2% (w/v) agarose gel electrophoresis.

### Reverse transcription-quantitative PCR (RT-qPCR)

Purified RNA (1 µg) was treated with DNase I to remove any genomic traces from the sample. 1 µl of 25 mM EDTA was added to the treated RNA followed by an incubation of 10 min at 65 °C to inactivate the DNase I. For reverse transcription, 1 µl of 50 µM oligodT, 1 µl of 50 µM random hexamers and 10 mM dNTPs were added to the treated RNA^83^. The sample was incubated at 65 °C for 5 min and then on ice for 2 min. 7 µl of reverse transcription master mix, comprising 5X first strand buffer, 0.1 M DTT, 40 U/µl RNaseOUT, and 200 U/µL Superscript III reverse transcriptase were added to the above RNA mix. The sample was then incubated for the following conditions to synthesize the first strand of cDNA: 50 °C for 60 min followed by 70 °C for 15 min. The cDNA product was diluted five-fold and stored at −20 °C until quantitative PCR (qPCR).

RT-qPCR reactions were performed in a 15-µl final volume with 7.5 µl of a SYBR master mix (Kappa, Sigma), 4.5 µl of water, 10 µM of each forward and reverse primer. Cycling conditions were 95 °C for 10 min hold, followed by 40 cycles of 95 °C for 10 s, 60 °C for 15 s and 72 °C for 20 s. A melt curve analysis was performed to determine the primer specificity with the following conditions: Ramping of temperature from 72–95 °C by one degree Celsius at each step, with a waiting time of 5 s after each ramping step. All the RT-qPCR reactions were run in triplicate for all the samples across the eight candidate reference genes with negative water and reverse transcriptase control using the RotorGene-Q (Qiagen, Germany).

### *In silico* identification, gene sequence search and primer design

Based on a literature search, eight candidate reference genes for each of *Chionochloa* and *Celmisia* were selected for the study. Reference gene sequences present in sunflower, *Chrysanthemum*, rice, and maize were used as query sequences to search for the orthologous sequences in *Celmisia* and *Chionochloa*. These query sequences were blasted against the transcriptome assembly of *Celmisia* and *Chionochloa* (unpublished data) to identify the candidate reference genes. The putative sequences were further analysed using multiple sequence alignment and phylogenetic analysis. Both the forward and reverse primers for each of the candidate reference genes were designed using Primer premier 6.0^84^ with the following conditions: Tm values ranging between 58–62 °C, GC content between 40–60%, primer lengths of 18–30 base-pair (bp), and product length of 100–300 bp. The primers were synthesized by the Macrogen Company (South Korea). All the primers were tested for amplification efficiency and coefficient of determination across all the samples using LinReg PCR software^85^. This software calculates the amplification efficiency based on the data points measured from the amplification curve when plotted against the log-florescence axis. The amplified products were run on 1.5% (w/v) agarose gel to verify the specificity of the primers along with dissociation curve analysis. The amplified products were cleaned up using a DNA agarose extraction kits (New England Biolabs) as per the manufacturer’s instructions for sequencing. These products were again sequenced using Sanger sequencing^86^ at the University of Canterbury, to confirm the identity of the amplified PCR product. The sequences have been lodged in GenBank. The Accession numbers are provided in Supplementary File 6.

### Determination of reference gene expression stability

The stability of expression of the reference genes across distinct temporal-spatially collected samples was assessed using five different statistical packages: geNorm, Normfinder, BestKeeper, ΔC_t_ and RefFinder. All the packages were used according to the manufacturer’s instructions. Averaged C_q_ values from the triplicate reactions for each sample and for each gene combination were used for the analysis. For geNorm and Normfinder, the raw C_q_ values were transformed to relative quantities (RQ) using the formula ${\rm{RQ}}={2}^{({{\rm{C}}}_{q}{\rm{\min }}-{{\rm{C}}}_{q}{\rm{sample}})}$, where C_q_ min is the lowest C_q_ value across the sample pool. The geNorm algorithm calculates the expression stability value (M) and pairwise variation (V). All the genes across different conditions were ranked by their stability value, the lower the value of M, the more stable the gene expression. The computed pairwise variation, based on the normalisation factor, allows for the determination of the minimum number of reference genes required for normalisation of the data. A value of V less than 0.15 indicates the appropriate number of reference genes required for analysis^55^. Normfinder evaluates the expression stability of candidate reference genes at inter-group and intra-group levels. Ideally, the two genes with the lowest stability values are the most appropriate genes to be used for normalisation^56^. BestKeeper analyses the candidate genes based on the standard deviation and coefficient of variation (CV) of the C_q_ values in a sample pool. The smaller the standard deviation and CV, and the higher the coefficient of regression, the more stable is the gene^57^. The ΔC_t_ method compares the relative expression of the pair of genes in a sample pool, suggesting a correlation between the variations in the C_q_ values of a candidate reference gene across the samples with their stability. RefFinder is another tool that determines a comprehensive ranking of the stability of the candidate reference genes based on the inputs from geNorm, Normfinder, Bestkeeper and ∆C_t_ methods. The tool analyses the output from all the statistical software and generates a comprehensive ranking list of reference genes based on their stability^59^.

### Validation of the reference gene analysis

*Cp Heading Date1* (*Hd1*) and *Cl CONSTANS* (*CO*) were used as the targets to validate the selected reference genes for data normalisation in *Chionochloa* and *Celmisia*, respectively. *CO* and *Hd1* are two orthologous proteins found in Arabidopsis and rice, respectively^66,87^. These proteins belong to the zinc-finger transcription factor family that upregulates the expression of florigen (*FT*) that codes for the flowering hormone^56^. These genes physically bind to the promoter of *FT* to induce flowering in plants. The expression analysis was done as described above and the results statistically tested using two-way analysis of variance (ANOVA).

### Ethics approval and consent to participate

All plant material was collected with appropriate permissions and consultation. Department of Conservation permit number 40225-FLO.

## Supplementary information

Supplementary data files

## Data Availability

The data generated and analysed is provided in the article and Supplementary Files. The accession numbers of the sequences are in Supplementary File 6.
