# Supplementary material for: Selection of reference genes for flowering pathway analysis in the masting plants, Celmisia lyallii and Chionochloa pallens, under variable environmental conditions
Source: Sci Rep. 2019 Jul 5;9:9767. doi: 10.1038/s41598-019-45780-1 (PMC6611903; doi:10.1038/s41598-019-45780-1)
Supplement: Supplementary file 1 — Supplementary data files [file 41598_2019_45780_MOESM1_ESM.pdf]

Selection of reference genes for flowering pathway analysis in the masting plants, *Celmisia lyallii* and *Chionochloa pallens*, under variable environmental conditions

Samarth[1] and Paula E. Jameson[1*]

[1] School of Biological Sciences, University of Canterbury, Christchurch, New Zealand

[*]corresponding author

Corresponding author

Paula E. Jameson

Professor Emerita

School of Biological Sciences, University of Canterbury, Christchurch, New Zealand

E-mail: paula.jameson@canterbury.ac.nz

Supplementary file 1

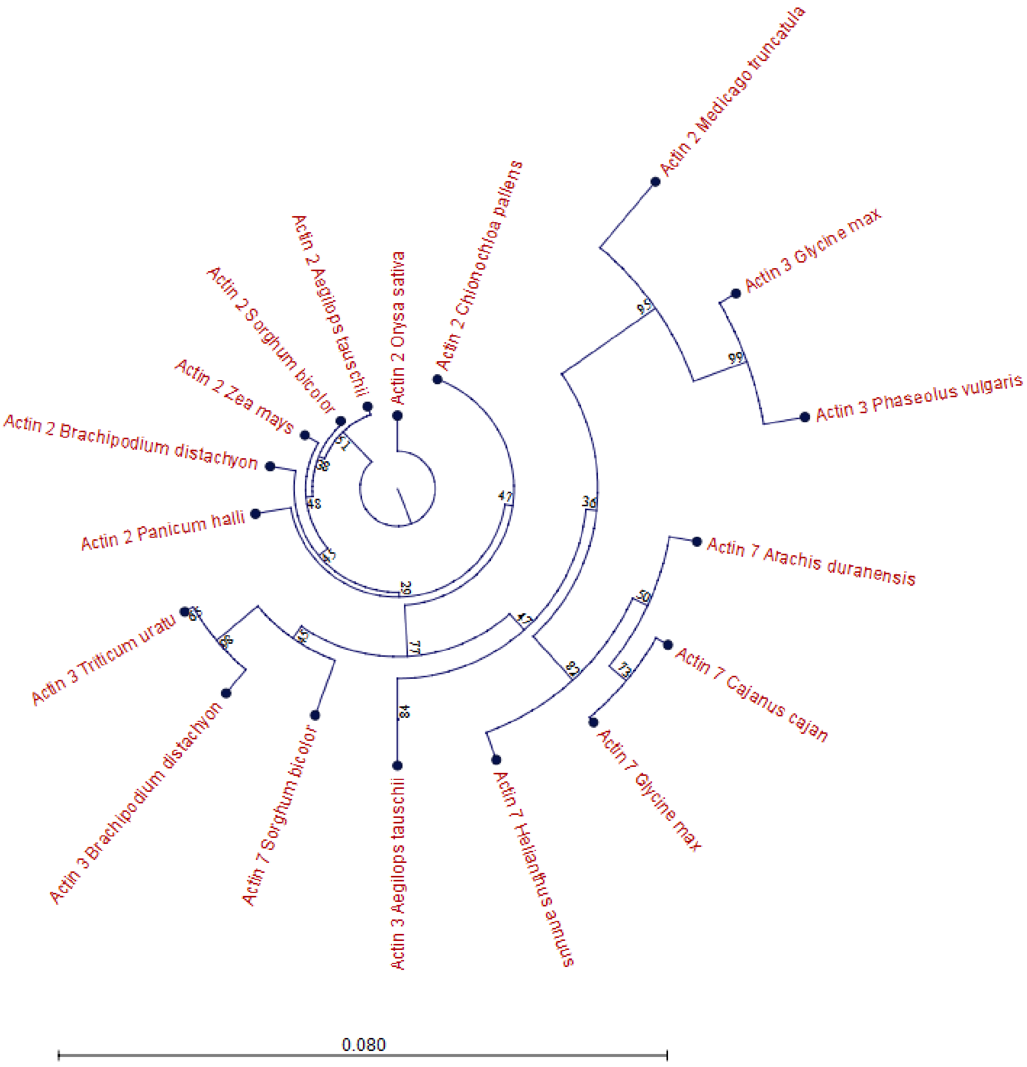

Phylogenetic tree of **Actin** protein family. The evolutionary history was inferred by using the Maximum Likelihood method based on the JTT matrix-based model. The bootstrap consensus tree inferred from 1000 replicates is taken to represent the evolutionary history of the taxa analysed. Branches corresponding to partitions reproduced in less than 50% bootstrap replicates are collapsed. The percentage of replicate trees in which the associated taxa clustered together in the bootstrap test (1000 replicates) are shown next to the branches. Initial tree(s) for the heuristic search were obtained by applying the Neighbor-Joining method to a matrix of pairwise distances estimated using a JTT model.

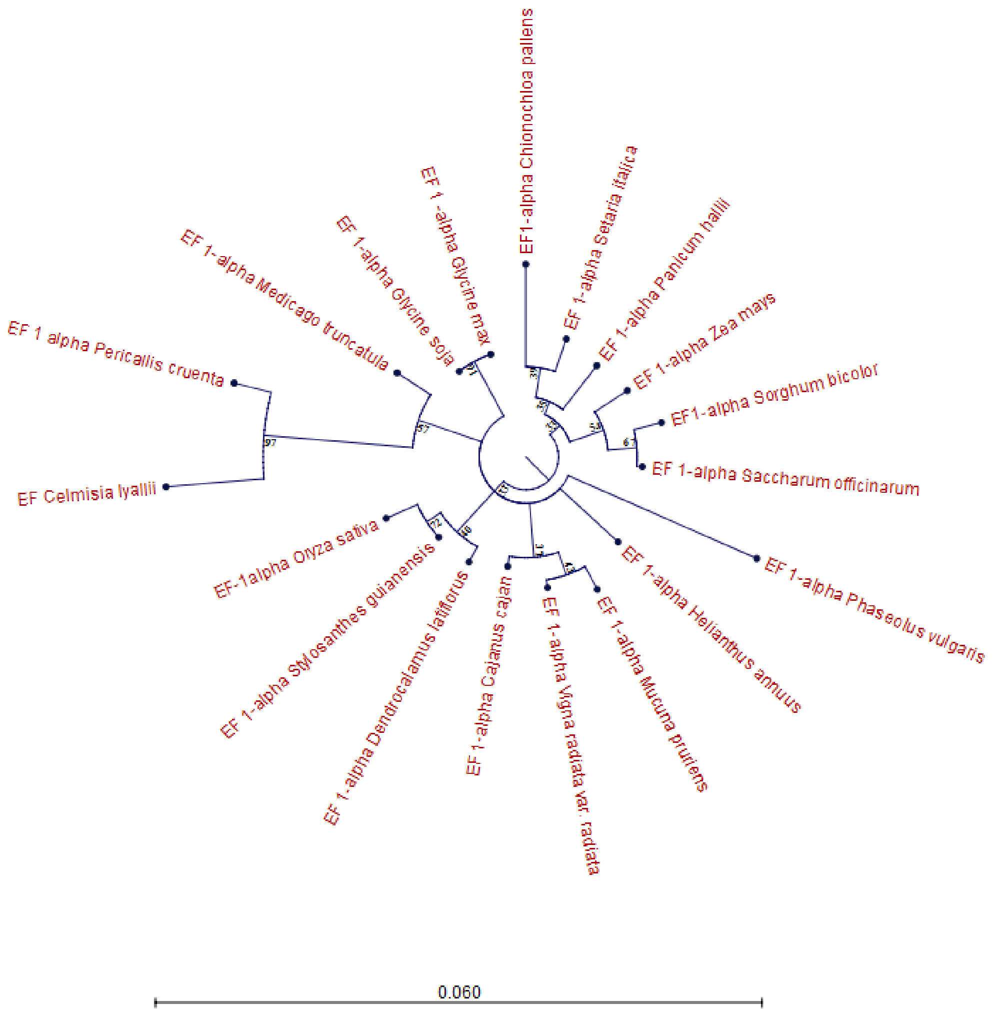

Phylogenetic tree of **Elongation factor 1-alpha** protein family. The evolutionary history was inferred by using the Maximum Likelihood method based on the JTT matrix-based model. The bootstrap consensus tree inferred from 1000 replicates is taken to represent the evolutionary history of the taxa analysed. Branches corresponding to partitions reproduced in less than 50% bootstrap replicates are collapsed. The percentage of replicate trees in which the associated taxa clustered together in the bootstrap test (1000 replicates) are shown next to the branches. Initial tree(s) for the heuristic search were obtained by applying the Neighbor-Joining method to a matrix of pairwise distances estimated using a JTT model.

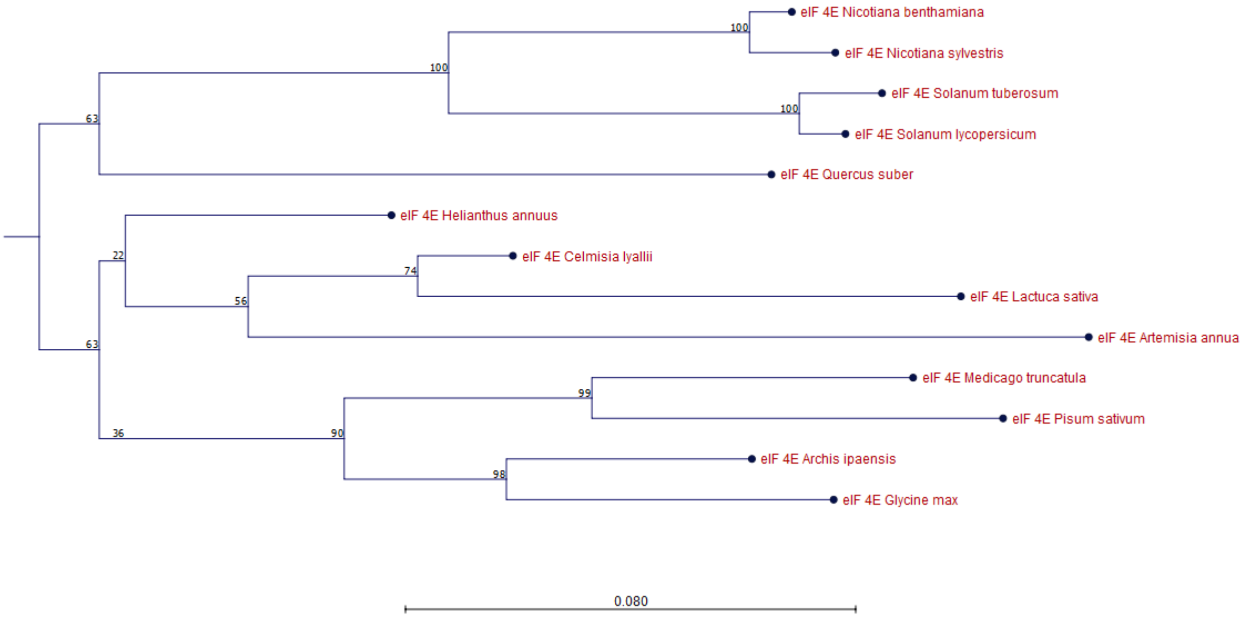

Phylogenetic tree of **eukaryotic Initiation factor 4ε** protein family. The evolutionary history was inferred by using the Maximum Likelihood method based on the JTT matrix-based model. The bootstrap consensus tree inferred from 1000 replicates is taken to represent the evolutionary history of the taxa analysed. Branches corresponding to partitions reproduced in less than 50% bootstrap replicates are collapsed. The percentage of replicate trees in which the associated taxa clustered together in the bootstrap test (1000 replicates) are shown next to the branches. Initial tree(s) for the heuristic search were obtained by applying the Neighbor-Joining method to a matrix of pairwise distances estimated using a JTT model.

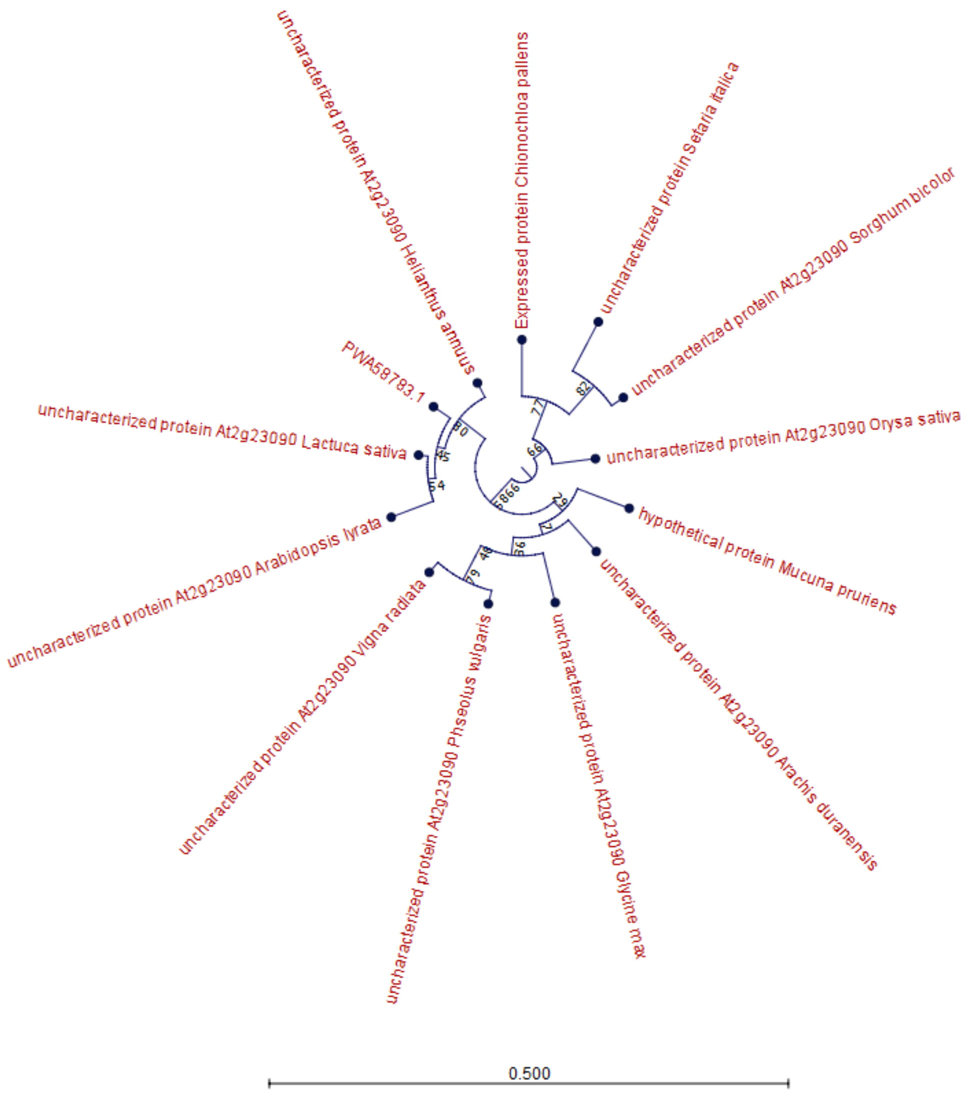

Phylogenetic tree of **expressed protein** family. The evolutionary history was inferred by using the Maximum Likelihood method based on the JTT matrix-based model. The bootstrap consensus tree inferred from 1000 replicates is taken to represent the evolutionary history of the taxa analysed. Branches corresponding to partitions reproduced in less than 50% bootstrap replicates are collapsed. The percentage of replicate trees in which the associated taxa clustered together in the bootstrap test (1000 replicates) are shown next to the branches. Initial tree(s) for the heuristic search were obtained by applying the Neighbor-Joining method to a matrix of pairwise distances estimated using a JTT model.

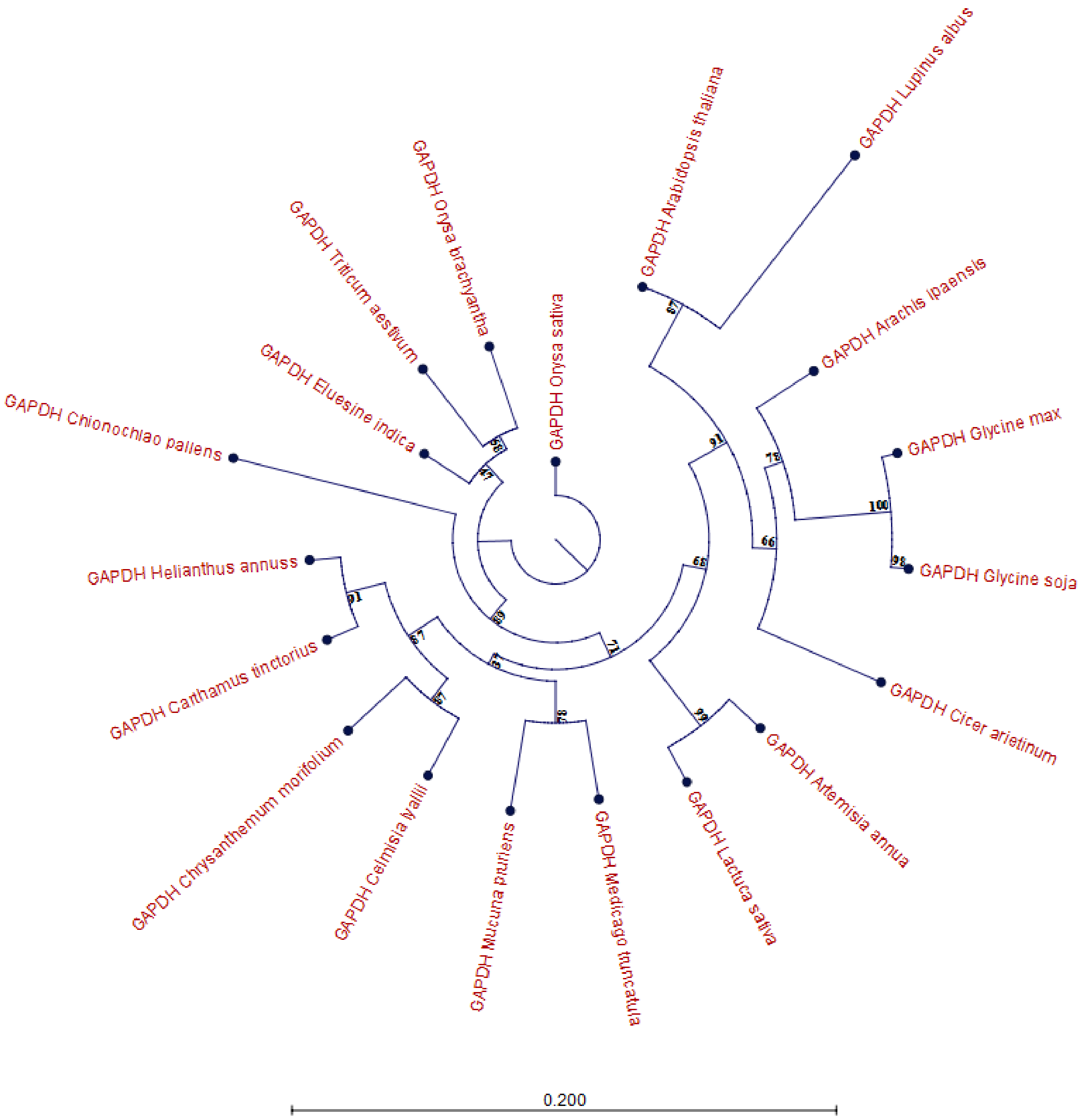

Phylogenetic tree of **GAPDH** protein family. The evolutionary history was inferred by using the Maximum Likelihood method based on the JTT matrix-based model. The bootstrap consensus tree inferred from 1000 replicates is taken to represent the evolutionary history of the taxa analysed. Branches corresponding to partitions reproduced in less than 50% bootstrap replicates are collapsed. The percentage of replicate trees in which the associated taxa clustered together in the bootstrap test (1000 replicates) are shown next to the branches. Initial tree(s) for the heuristic search were obtained by applying the Neighbor-Joining method to a matrix of pairwise distances estimated using a JTT model.

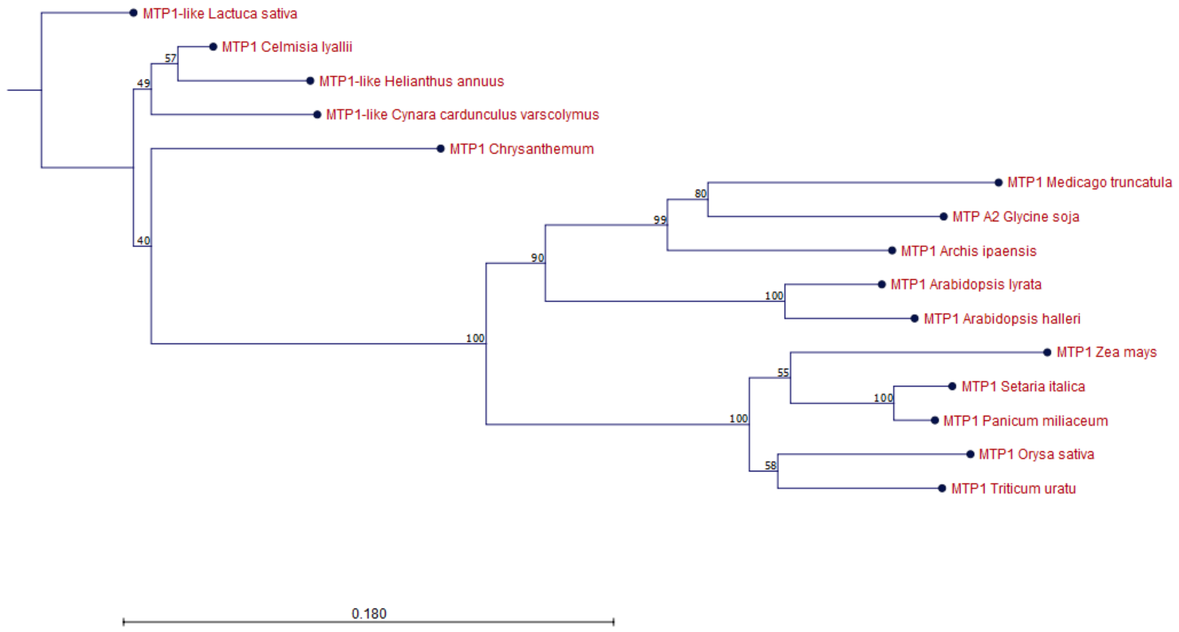

Phylogenetic tree of **MTP** protein family. The evolutionary history was inferred by using the Maximum Likelihood method based on the JTT matrix-based model. The bootstrap consensus tree inferred from 1000 replicates is taken to represent the evolutionary history of the taxa analysed. Branches corresponding to partitions reproduced in less than 50% bootstrap replicates are collapsed. The percentage of replicate trees in which the associated taxa clustered together in the bootstrap test (1000 replicates) are shown next to the branches. Initial tree(s) for the heuristic search were obtained by applying the Neighbor-Joining method to a matrix of pairwise distances estimated using a JTT model.

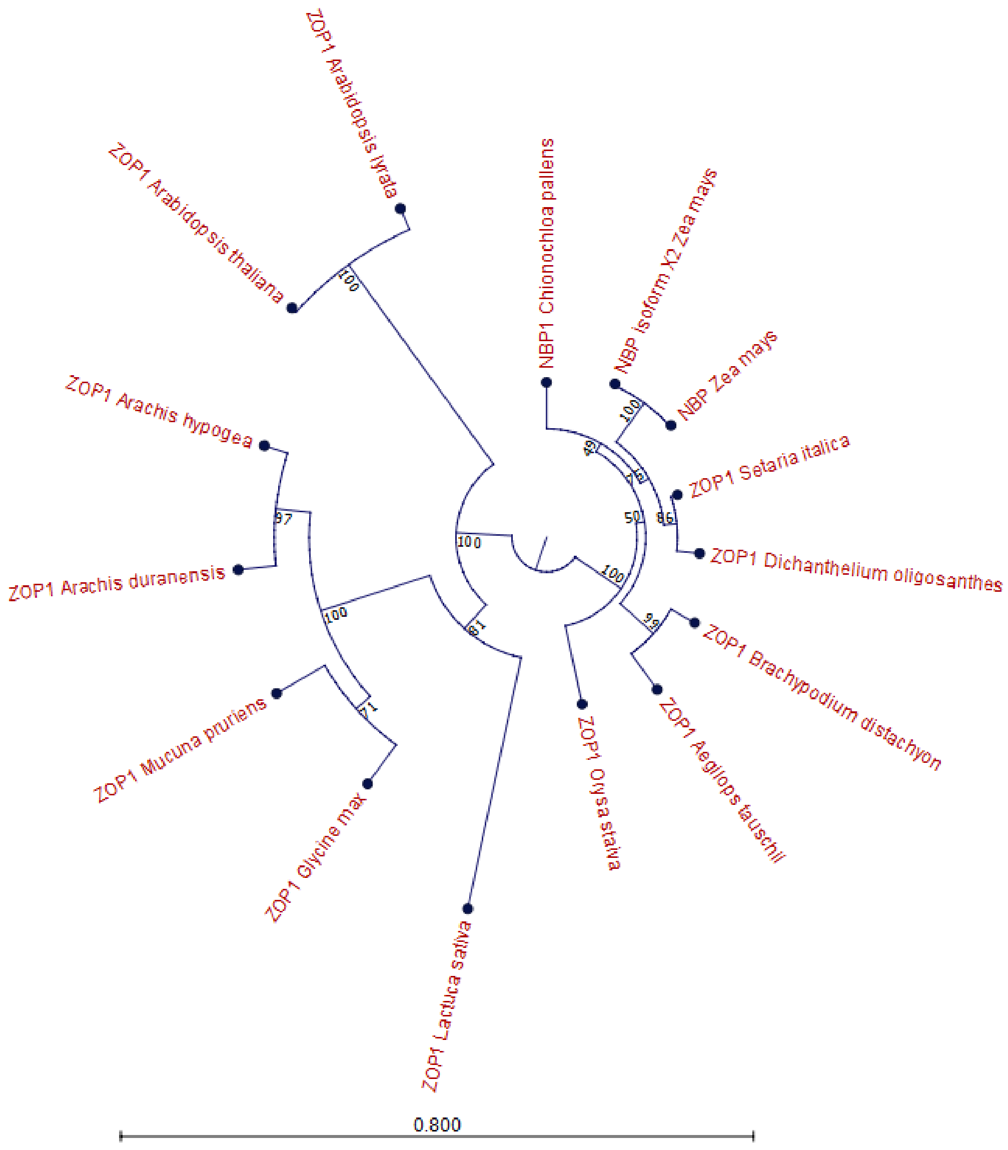

Phylogenetic tree of **NBP** protein family. The evolutionary history was inferred by using the Maximum Likelihood method based on the JTT matrix-based model. The bootstrap consensus tree inferred from 1000 replicates is taken to represent the evolutionary history of the taxa analysed. Branches corresponding to partitions reproduced in less than 50% bootstrap replicates are collapsed. The percentage of replicate trees in which the associated taxa clustered together in the bootstrap test (1000 replicates) are shown next to the branches. Initial tree(s) for the heuristic search were obtained by applying the Neighbor-Joining method to a matrix of pairwise distances estimated using a JTT model.

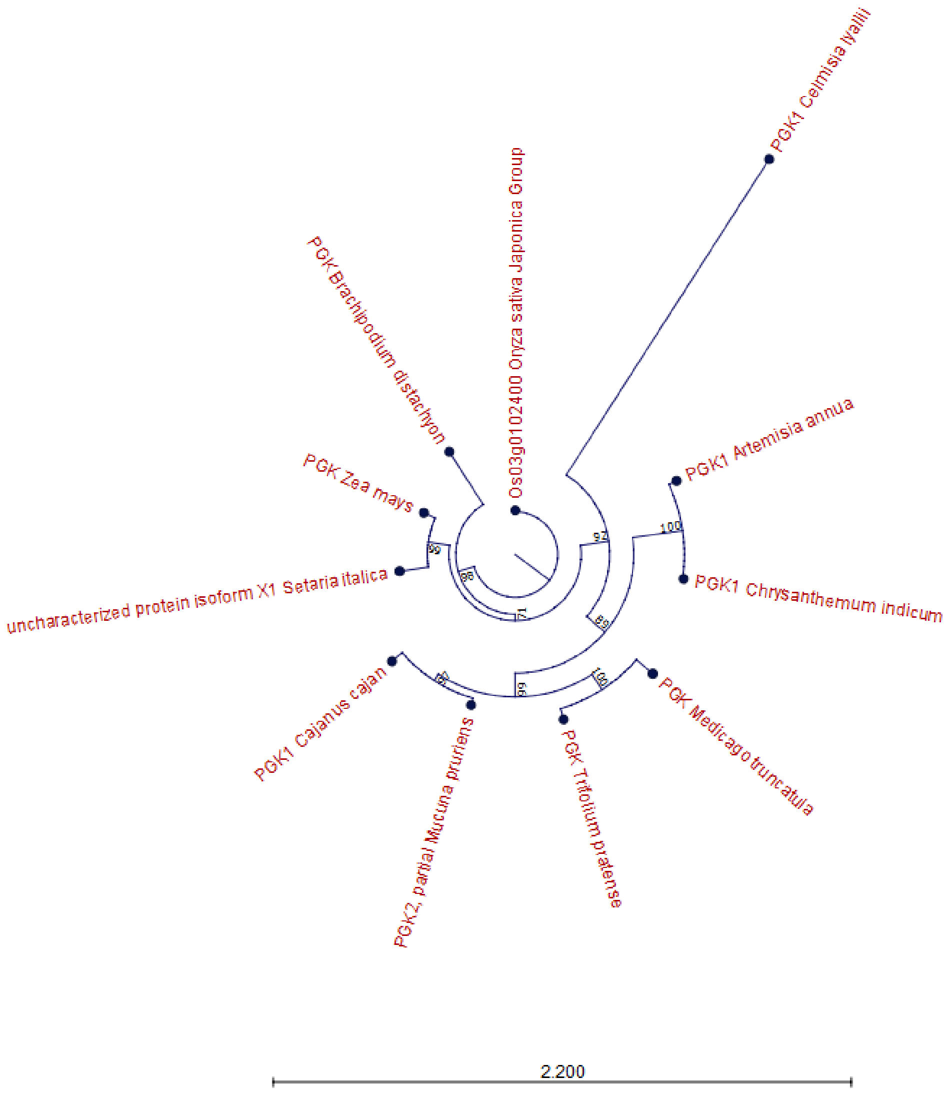

Phylogenetic tree of **PGK** protein family. The evolutionary history was inferred by using the Maximum Likelihood method based on the JTT matrix-based model. The bootstrap consensus tree inferred from 1000 replicates is taken to represent the evolutionary history of the taxa analysed. Branches corresponding to partitions reproduced in less than 50% bootstrap replicates are collapsed. The percentage of replicate trees in which the associated taxa clustered together in the bootstrap test (1000 replicates) are shown next to the branches. Initial tree(s) for the heuristic search were obtained by applying the Neighbor-Joining method to a matrix of pairwise distances estimated using a JTT model.

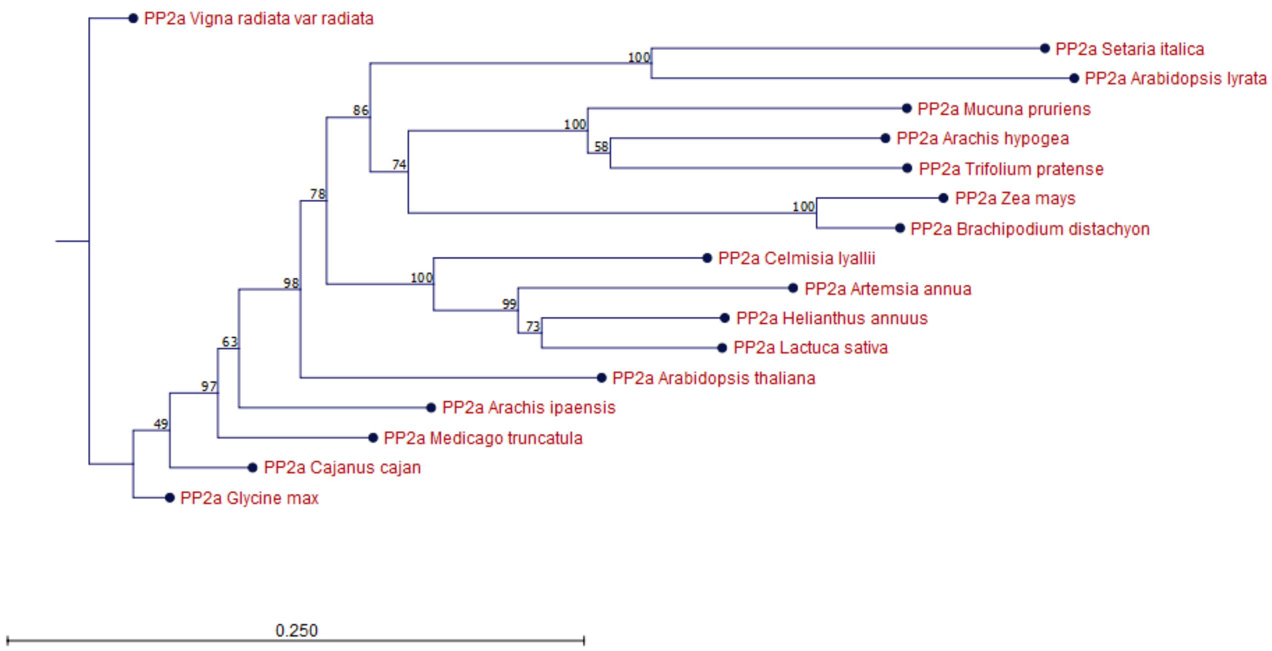

Phylogenetic tree of **PP2a** protein family. The evolutionary history was inferred by using the Maximum Likelihood method based on the JTT matrix-based model. The bootstrap consensus tree inferred from 1000 replicates is taken to represent the evolutionary history of the taxa analysed. Branches corresponding to partitions reproduced in less than 50% bootstrap replicates are collapsed. The percentage of replicate trees in which the associated taxa clustered together in the bootstrap test (1000 replicates) are shown next to the branches. Initial tree(s) for the heuristic search were obtained by applying the Neighbor-Joining method to a matrix of pairwise distances estimated using a JTT model.

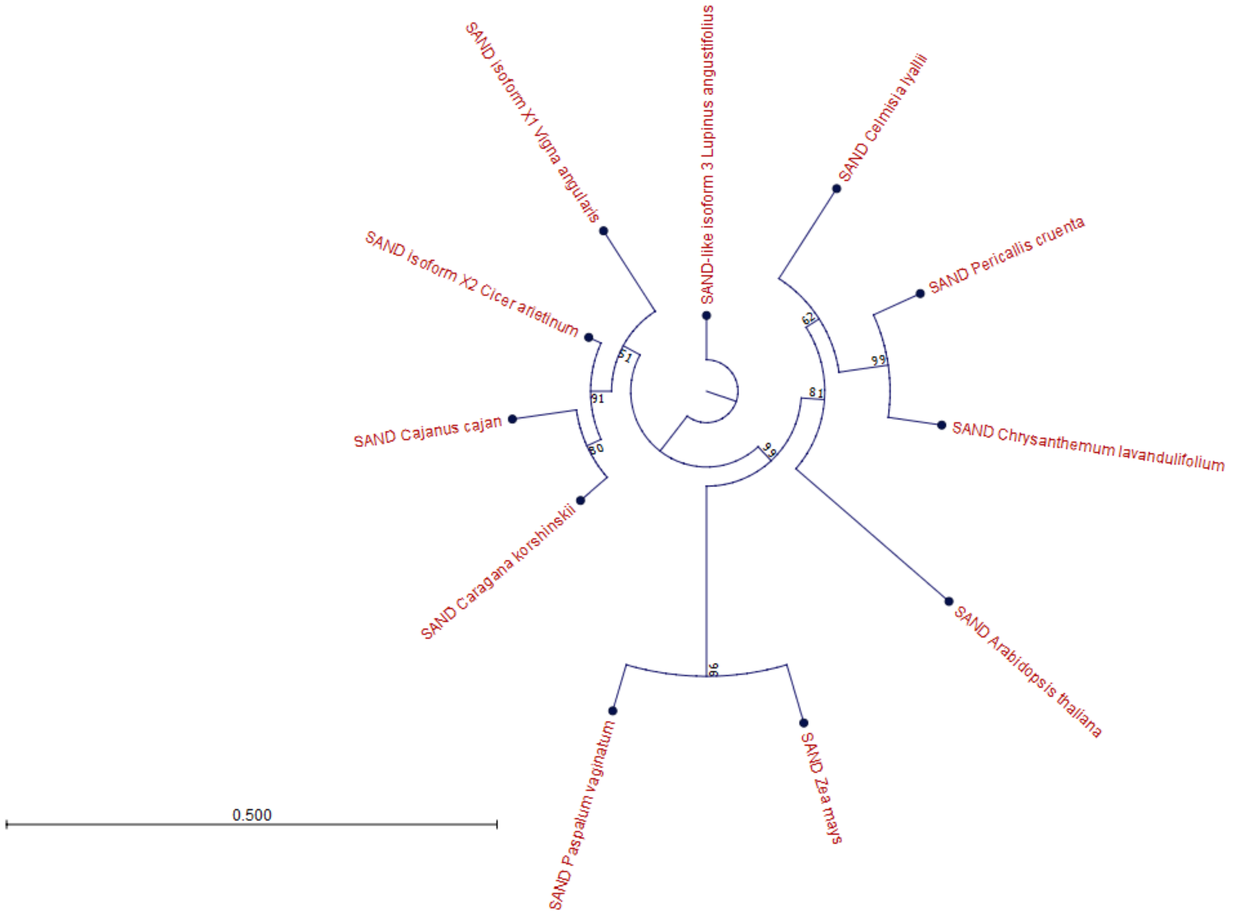

Phylogenetic tree of **SAND** protein family. The evolutionary history was inferred by using the Maximum Likelihood method based on the JTT matrix-based model. The bootstrap consensus tree inferred from 1000 replicates is taken to represent the evolutionary history of the taxa analysed. Branches corresponding to partitions reproduced in less than 50% bootstrap replicates are collapsed. The percentage of replicate trees in which the associated taxa clustered together in the bootstrap test (1000 replicates) are shown next to the branches. Initial tree(s) for the heuristic search were obtained by applying the Neighbor-Joining method to a matrix of pairwise distances estimated using a JTT model.

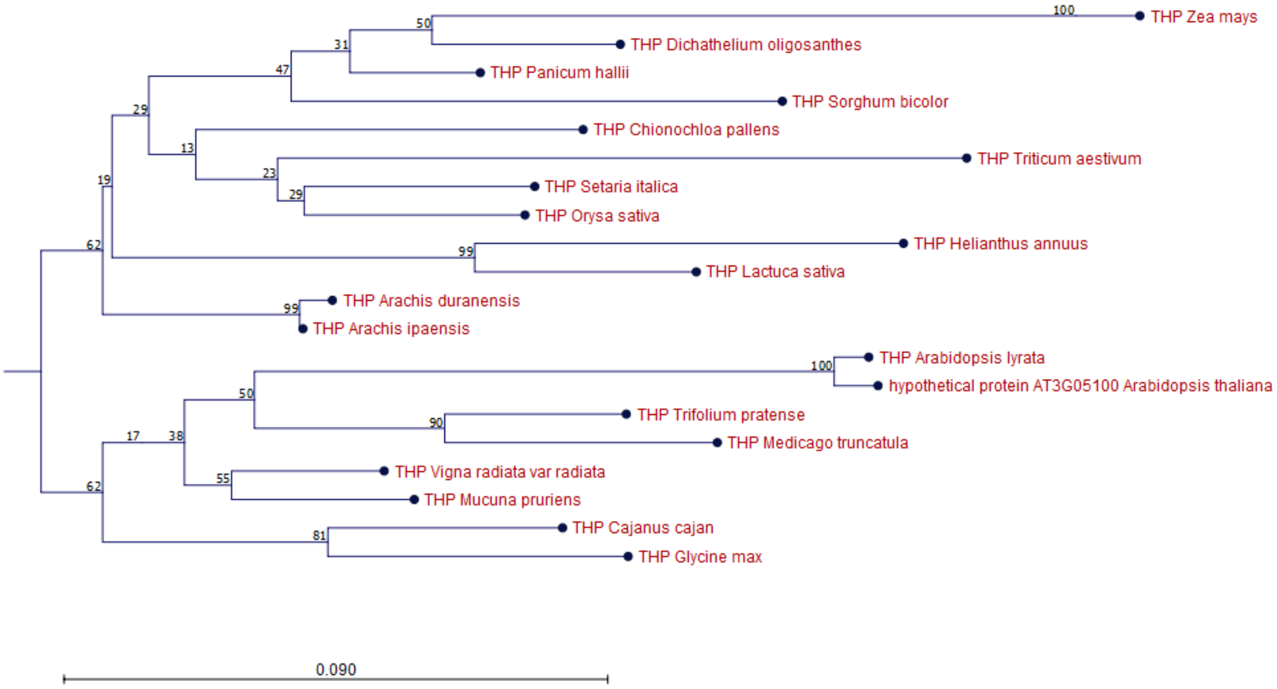

Phylogenetic tree of **THP** protein family. The evolutionary history was inferred by using the Maximum Likelihood method based on the JTT matrix-based model. The bootstrap consensus tree inferred from 1000 replicates is taken to represent the evolutionary history of the taxa analysed. Branches corresponding to partitions reproduced in less than 50% bootstrap replicates are collapsed. The percentage of replicate trees in which the associated taxa clustered together in the bootstrap test (1000 replicates) are shown next to the branches. Initial tree(s) for the heuristic search were obtained by applying the Neighbor-Joining method to a matrix of pairwise distances estimated using a JTT model.

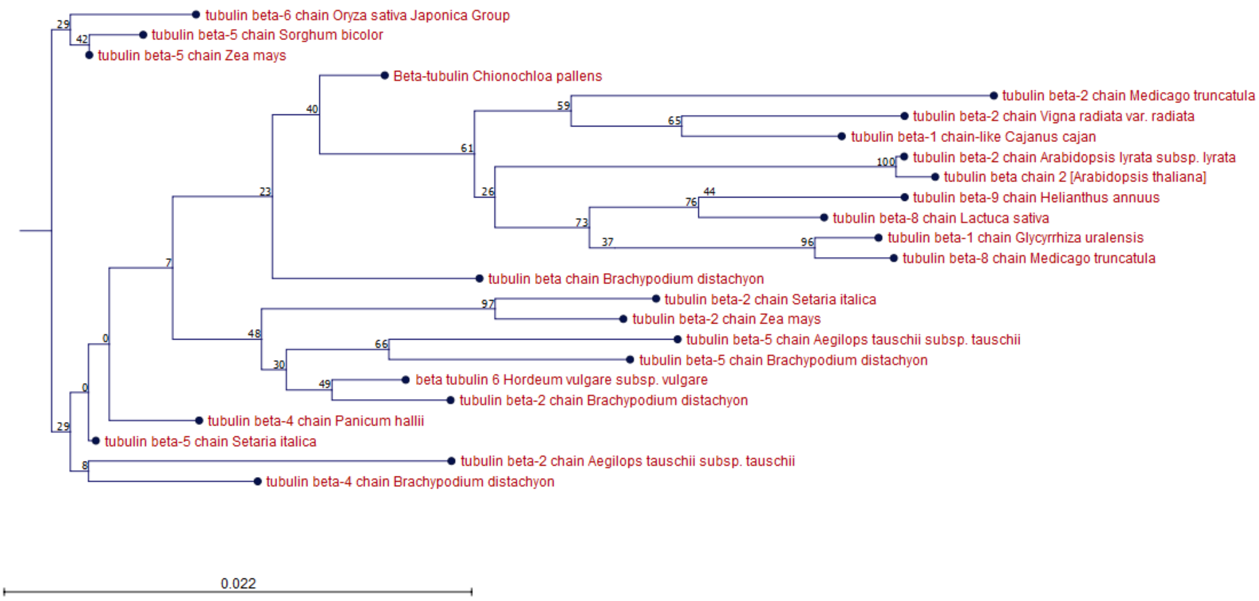

Phylogenetic tree of **Tubulin** protein family. The evolutionary history was inferred by using the Maximum Likelihood method based on the JTT matrix-based model. The bootstrap consensus tree inferred from 1000 replicates is taken to represent the evolutionary history of the taxa analysed. Branches corresponding to partitions reproduced in less than 50% bootstrap replicates are collapsed. The percentage of replicate trees in which the associated taxa clustered together in the bootstrap test (1000 replicates) are shown next to the branches. Initial tree(s) for the heuristic search were obtained by applying the Neighbor-Joining method to a matrix of pairwise distances estimated using a JTT model.

Supplementary file S2

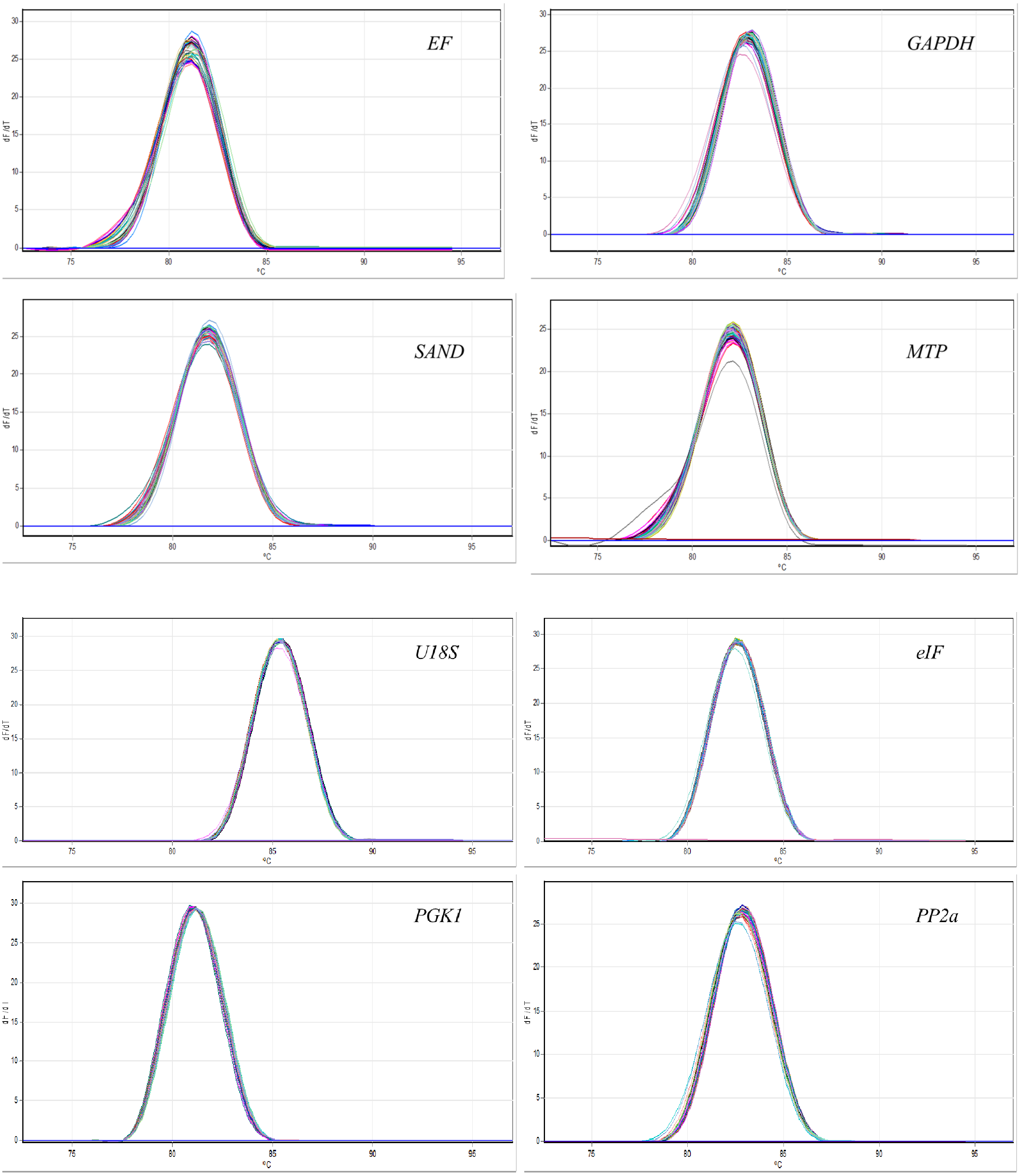

Figure S2a: Melt curve analysis for candidate reference genes in *Celmisia*.

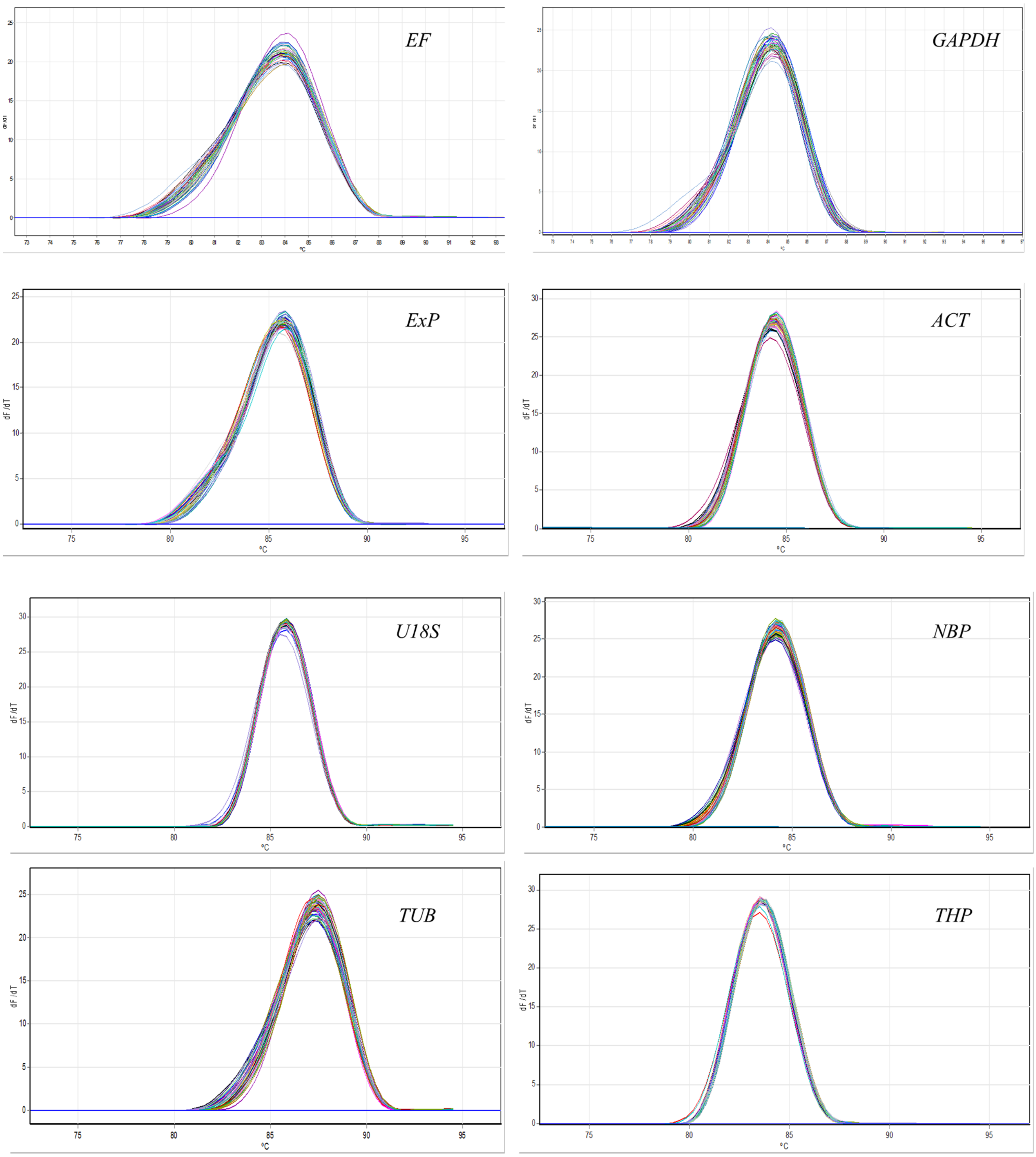

Figure S2b: Melt curve analysis for candidate reference genes in *Chionochloa*.

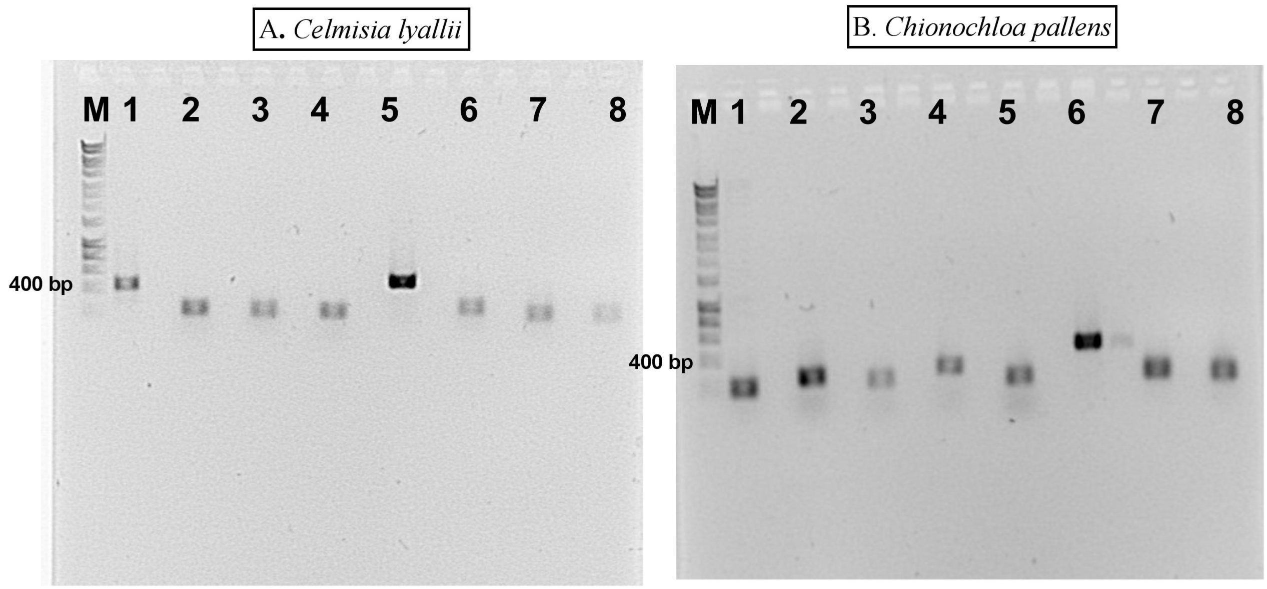

Supplementary figure S3. Agarose gel electrophoresis (1.5%) showing amplified PCR products from a) *Celmisia lyallii*: 1-*EF,* 2-*PP2a*, 3- *PGK1*, 4-*eIF*, 5-*18S*, 6-*GAPDH*, 7-*SAND*, 8-*MTP* b) *Chionochloa pallens*: 1-*Tub,* 2-*EF*, 3- *NBP*, 4-*THP*, 5-*Act*, 6-*18S*, 7-*ExP*, 8-*GAPDH* along with marker (M).

Supplementary file S4

Raw Cq values of identified reference genes from *Chionochloa pallens*

	EF			gapdh			u18s			ACT			ExP			NBP			THP			B-TUB		
UC early flw																								
Sample no	Rep 1	Rep 2	Rep 3	Rep 1	Rep 2	Rep 3	Rep 1	Rep 2	Rep 3	Rep 1	Rep 2	Rep 3	Rep 1	Rep 2	Rep 3	Rep 1	Rep 2	Rep 3	Rep 1	Rep 2	Rep 3	Rep 1	Rep 2	Rep 3
Jan-16	16.005	16.085	15.99	18.94	18.98	18.65	11.055	11.185	10.885	20.59	20.59	19.52	17.945	18.185	18.04	23.32	23.1375	23.26	17.27	17.18	17.38	20.09	20.9	20.795
Feb-16	16.58	16.75	16.64	19.515	19.465	19.315	11.04	11.165	10.945	20.87	20.88	19.96	18.12	18.19	18.27	23.755	23.57	23.55	16.99	16.975	17.03	20.8	21.81	21.94
May-16	18.12	18.1	18.245	18.93	18.9	19.045	12.69	12.88	12.495	23.145	23.245	22.135	18.52	18.445	18.42	23.78	23.6675	23.81	17.325	17.31	17.285	21.95	22.81	22.845
Sep-16	15.765	16.835	16.77	19.645	19.63	19.675	9.63	9.57	9.53	20.67	20.56	19.45	17.825	17.925	17.915	23.035	22.8425	22.835	16.7	16.575	16.65	19.945	20.66	20.885
Mar-17	16.69	16.88	17.11	19.52	19.71	19.535	10.135	10.175	9.97	22.755	22.45	22.045	18.32	18.15	18.12	22.71	22.7525	22.01	17.28	17.465	17.415	20.985	21.77	21.755
UC veg																								
Jan-16	16.325	16.235	16.305	19.305	19.28	19.155	11.465	11.45	11.225	20.665	20.63	19.37	17.565	17.54	17.58	23.16	22.8375	23.035	16.59	16.595	16.57	20.74	21.685	21.7
Feb-16	17.61	17.79	17.74	21.205	21.225	21.135	11.865	11.965	11.7	21.32	21.305	20.305	18.635	18.475	18.41	23.925	23.755	23.855	16.82	16.855	16.725	21.65	22.77	22.635
May-16	16.74	17.07	17.11	18.47	18.54	18.59	12.025	12.055	11.765	21.77	21.95	20.865	18.055	18.24	18.15	23.235	22.915	23.18	16.54	16.59	16.575	20.81	21.895	21.93
Sep-16	16.32	16.425	16.48	18.62	18.425	18.365	9.04	9.075	8.84	20.89	20.83	19.575	17.75	17.99	18.01	23.25	22.97	23.07	16.735	16.895	16.9	20.555	21.81	21.715
Mar-17	16.115	16.3	16.235	18.5	18.56	18.145	9.7	9.595	9.46	20.685	20.81	19.575	17.105	17.41	17.36	23.545	23.195	23.195	16.22	16.355	16.385	20.93	21.93	21.815
UC flw																								
Jan-16	15.925	16.075	15.93	19.165	19.13	19.265	12.335	12.47	12.195	20.205	20.415	19.26	18.21	18.23	18.365	23.47	23.14	23.37	17.855	17.97	18.23	20.805	21.525	21.495
Feb-16	17.01	17.455	17.43	20.6	20.42	20.73	11.42	11.53	11.31	21.565	21.405	20.31	18.37	18.56	18.365	23.93	23.445	23.925	17.95	18.015	18.205	22.13	23.005	22.985
May-16	16.565	16.725	16.65	18.45	18.53	18.29	9.155	9.225	8.88	21.21	20.99	20.025	18.42	18.53	18.305	23.255	22.875	23.01	17.285	17.34	17.425	20.64	21.675	21.68
Sep-16	16.265	16.295	16.395	18.045	18.13	18.09	9.11	9.27	8.83	20.895	20.91	19.615	18.075	17.995	18.395	23.025	22.595	22.82	17.26	17.395	17.375	20.53	21.505	21.575
Mar-17	16.415	16.585	16.38	18.8	18.92	18.725	10.935	11.055	10.77	21.455	21.445	20.32	16.99	16.935	17.345	23.54	23.22	23.53	16.495	16.495	16.465	21.43	22.32	22.205
1070 veg																								
Jan-16	20.055	20.83	20.765	21.305	21.045	21.13	9.41	9.44	9.175	24.09	24.055	22.87	19.495	19.325	19.315	23.83	23.675	23.645	19.275	19.24	19.14	22.965	23.72	23.7
Mar-16	16.245	16.365	16.4	19.37	19.165	19.335	8.76	8.72	8.57	20.52	20.555	19.475	18	17.885	17.78	23.215	23.005	23.185	16.43	16.5	16.375	20.325	21.175	21.27
Oct-16	16.115	16.665	16.56	18.575	18.25	18.225	10.13	10.125	9.965	20.555	20.205	19.39	17.85	17.91	17.885	23.03	22.735	22.78	17.215	17.275	17.2	19.985	21.015	20.865
May-17	16.315	16.255	16.07	18.505	18.215	18.3	10.015	10.15	9.835	21.05	20.845	19.91	17.695	17.64	17.865	22.76	22.59	22.66	16.465	16.695	16.5	20.125	21.185	21.145
1520 flw																								
Jan-16	17.585	18.195	17.625	20.165	20.145	19.915	8.42	8.32	8.21	22.25	22.41	21.215	19.06	19.045	19.13	23.695	23.5375	23.38	18.285	18.325	18.305	21.625	22.465	22.675
Mar-16	16.12	16.575	16.51	18.98	19.08	18.9	8.865	8.795	8.625	21.06	20.945	19.92	18.015	18.135	18.07	23.5	23.225	23.14	16.73	16.67	16.765	18.84	20.07	19.98
Oct-16	17.51	17.845	17.915	19.3	19.86	19.355	8.695	8.61	8.47	21.96	21.82	21.28	19.51	19.48	19.625	22.06	22.0225	21.385	18.27	18.46	18.415	19.97	20.84	20.675
May-17	15.86	16.115	15.955	18.575	18.32	18.295	8.915	8.92	8.65	21.52	21.42	20.9	17.845	17.87	17.865	21.4	21.3575	20.665	16.58	16.805	16.67	19.635	20.745	20.77
1520 veg																								
Jan-16	17.13	17.25	16.98	19.43	19.355	19.385	10.35	10.425	10.2	21.835	21.695	20.715	18.475	18.525	18.6	22.95	22.9275	23.17	17.37	17.475	17.615	20.73	21.77	21.575
Mar-16	14.825	15.245	15.04	17.36	17.295	17.425	8.265	8.235	7.97	19.505	19.79	18.455	16.925	16.8	16.905	21.885	21.75	21.72	15.925	15.525	15.865	18.955	20	20.06
Oct-16	15.835	15.965	15.96	17.62	17.585	17.17	9.575	9.665	9.4	21.345	21.18	20.85	17.645	17.475	17.52	21.595	21.5625	20.965	16.62	16.34	16.49	19.225	20.16	20.21
May-17	14.95	15.465	15.495	17.19	17.48	17.6	9.01	8.965	8.865	20.585	20.45	19.855	17.29	17.33	17.365	20.785	20.7925	20.25	16.33	16.19	16.31	19.105	20.22	20.11

Raw Cq values of identified reference genes from *Celmisia lyallii*

	EF			gapdh			Eif			u18s			SAND			MTP			PGK1			PP2A		
UC flw																								
Sample no	Rep 1	Rep 2	Rep 3	Rep 1	Rep 2	Rep 3	Rep 1	Rep 2	Rep 3	Rep 1	Rep 2	Rep 3	Rep 1	Rep 2	Rep 3	Rep 1	Rep 2	Rep 3	Rep 1	Rep 2	Rep 3	Rep 1	Rep 2	Rep 3
Jan/Feb-16	18.975	18.975	18.935	20.78	21.07	21.07	22.37	22.26	22	9.37	9.235	9.23	21.875	22.36	21.62	22.14	22.06	21.37	23.135	22.81	22.975	21.18	21.05	20.855
Mar-16	17.67	18.635	18.655	19.875	20.04	20.145	21.175	21.415	21.1	9.475	9.285	9.275	21.78	21.24	21.135	21.075	21.32	21.07	22.26	22.3	22.375	21.06	20.815	20.585
Oct-16	18.795	19.58	19.545	20.345	20.6	20.655	21.685	21.985	21.695	9.35	9.16	9.245	22.265	22.12	21.73	22.28	22.13	21.5	22.705	22.705	22.915	21.41	21.28	21.03
Mar-17	17.6	19.885	19.835	20.75	20.92	20.255	22.115	22.165	21.945	10.56	10.2	10.28	20.915	21.345	21.235	21.98	21.715	21.48	22.185	22.55	22.635	21.37	21.085	20.86
UC veg																								
Jan/Feb-16	18.285	18.345	17.84	19.5	20.69	20.565	22.22	22.3	22.025	9.8	9.68	9.72	22.3	21.885	21.8	20.96	21.445	21.21	22.54	22.755	22.825	21.07	21.055	20.675
Mar-16	18.925	18.855	18.81	19.65	19.77	20.05	21.41	21.345	21.125	9.725	9.57	9.61	21.69	21.455	20.905	20.62	21.225	21.05	21.95	22.035	22.115	20.775	20.555	20.3
Oct-16	17.795	19.05	19.17	19.48	20.005	20.1	21.28	21.535	21.34	9.95	9.8	9.89	22.09	21.755	21.045	21.03	21.34	20.785	22.055	22.115	22.38	20.83	20.82	20.55
Mar-17	18.865	19.89	19.88	20.95	20.665	20.505	22.395	22.185	22.165	10.55	10.415	10.325	22.9	22.315	21.955	20.995	21.145	20.915	22.695	22.675	22.83	21.49	21.2	21.145
1350 undug control veg																								
Jan/Feb-16	19.455	19.79	20.015	21.075	21.235	21.105	22.365	22.575	22.515	11.66	11.325	11.13	23.205	23.085	22.92	21.36	21.325	20.805	25.105	24.955	25.03	21.295	21.265	20.955
Mar-16	18.675	19.635	19.835	20.165	20.115	20.41	21.74	22.07	21.855	9.165	8.975	9.1	22.535	21.905	21.82	20.795	21.09	20.535	22.61	22.54	22.63	20.995	20.775	20.58
Oct-16	19.44	19.695	20.38	19.935	20.115	20.22	22.03	21.985	21.895	9.65	9.505	9.58	22.375	21.965	21.755	21.12	21.56	20.78	22.34	22.195	22.32	20.68	20.91	20.725
Mar-17	19.555	20.595	20.955	20.23	20.295	20.46	21.85	21.91	21.7	12.02	12.08	12.095	21.795	21.355	21.35	20.63	21.025	20.505	22.2	22.275	22.265	20.89	20.74	20.53
1350 dug control veg																								
Jan/Feb-16	20.3	19.765	20.105	21.66	21.32	21.34	21.95	21.975	21.81	17.56	17.45	17.425	23.505	23.625	23.16	20.95	21.505	20.93	22.835	22.8	22.96	21.255	21.23	20.95
Mar-16	19.11	18.66	19.59	19.565	19.93	19.875	21.33	21.52	21.305	10.175	9.975	9.985	22.01	21.745	21.28	20.54	20.67	20.035	22.505	22.055	22.13	20.635	20.125	20.245
Oct-16	19.085	19.625	19.72	18.83	19.705	19.895	21.625	21.335	21.395	10.075	9.925	9.955	21.79	21.605	21.115	20.41	20.41	20.2	21.74	21.635	21.86	20.385	20.54	20.295
Mar-17	17.98	18.51	20.02	19.565	20.045	20.18	21.505	21.625	21.4	11.21	11.035	11.015	21.885	21.255	21.31	20.85	20.745	20.41	22.335	22.005	22.275	20.83	20.685	20.33
1520 veg																								
Jan/Feb-16	20.475	20.69	20.33	20.555	20.595	20.51	21.55	21.71	21.395	15.8	15.395	15.53	22.57	22.4	21.935	20.53	20.72	20.295	22.99	22.695	22.81	21.615	21.325	20.785
Mar-16	19.355	19.725	19.695	20.2	20.42	20.42	21.625	21.615	21.42	11.285	10.945	10.98	22.255	21.97	21.67	20.42	21.095	20.535	22.405	22.29	22.11	20.615	20.64	20.465
Oct-16	19.415	19.54	19.61	20.25	20.365	20.75	21.43	21.83	21.35	8.535	8.44	8.44	22.14	21.635	21.51	20.195	20.705	20.445	21.775	21.89	21.935	20.93	20.61	20.41
Mar-17	18.455	18.89	19.295	19.43	20.645	20.44	21.695	21.8	21.565	11.17	11.175	10.85	22.14	21.475	21.23	20.66	20.34	20.42	22.15	22.32	22.155	20.375	20.69	20.52
1070 veg																								
Jan/Feb-16	18.89	19.29	19.485	19.87	20.31	20.3	21.74	21.855	21.56	10.235	10.45	10.15	22.615	22.37	22.045	21.31	21.185	20.85	23.135	22.75	23.1	20.745	20.72	20.375
Mar-16	18.245	19.82	19.77	19.625	20.38	20.365	21.57	21.585	21.345	11.63	11.4	11.205	22.06	21.66	21.755	20.02	20.68	20.415	21.835	21.985	22.095	20.58	20.485	20.095
Oct-16	19.155	19.485	20.7	18.67	19.4	19.645	20.72	20.955	20.735	9.955	9.83	9.845	21.485	21.365	20.97	19.425	20.1	19.805	20.955	21.235	21.245	19.955	19.795	19.515
Mar-17	19.405	19.5	19.64	19.785	19.96	20.155	21.82	21.845	21.345	9.01	8.955	8.675	22.07	21.87	21.585	20.225	21.1	20.595	22.03	22.025	22.08	20.775	20.64	20.21

Supplementary file S5

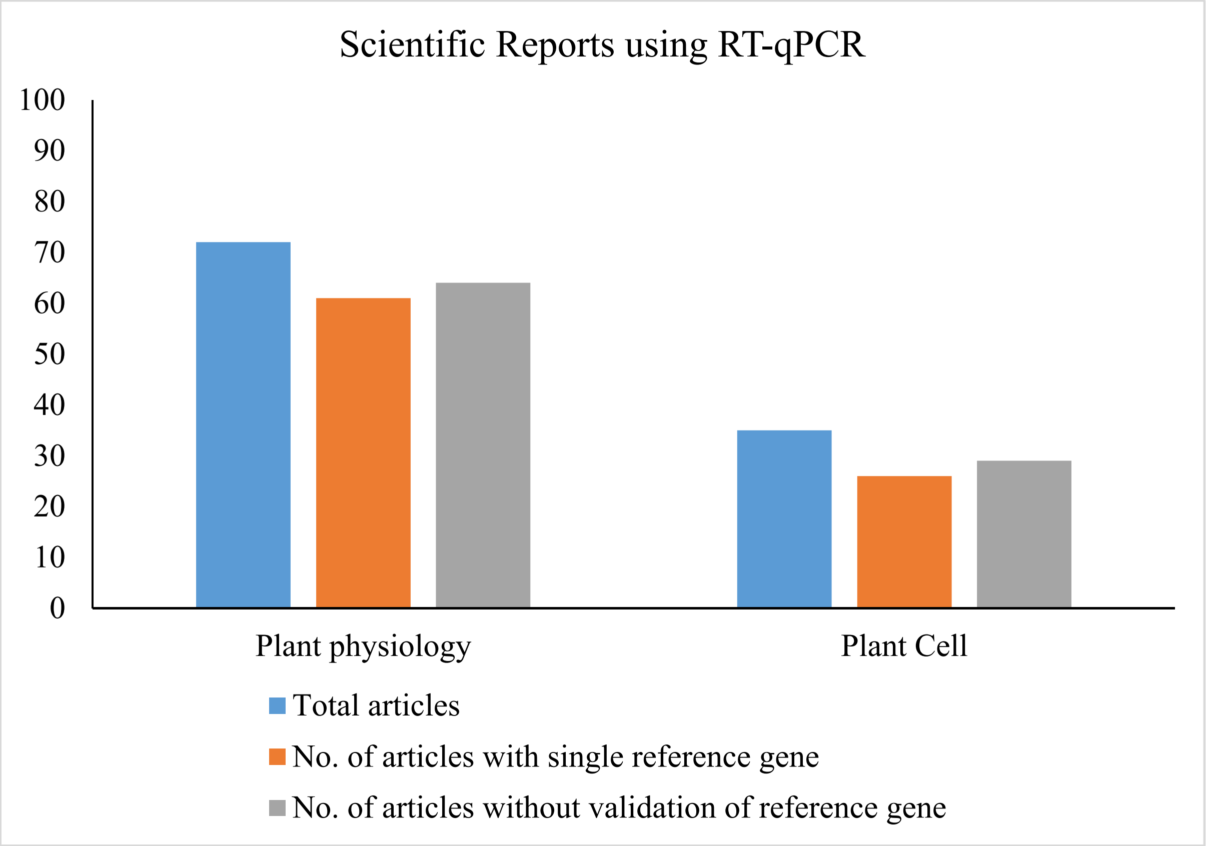

Figure S5: Scientific reports published in “Plant Cell” and “Plant Physiology” in past six months using RT-qPCR. The graph shows the total number of articles published, number of articles using one reference gene, and number of articles using invalidated reference gene(s) for their study. This graph shows an urgent need for systematic validation of reference gene serving as normalisation factor to achieve better accuracy to gene expression data analysis.

Supplementary file S6: Accession numbers for the sequences used in this study

Celmisia lyallii EF1-alpha - MN105994
Celmisia lyallii PP2a - MN105995
Celmisia lyallii GAPDH - MN105996
Celmisia lyallii eIF4-e - MN105997
Celmisia lyallii MTP -MN105998
Celmisia lyallii PGK1- MN105999
Celmisia lyallii SAND- MN106000

Chionochloa pallens Elongation factor 1-alpha (EF1-alpha) - MN106001
Chionochloa pallens Glyceraldehyde-6-phosphate dehydrogenase (GAPDH) - MN106002
Chionochloa pallens Actin (Act) - MN106003
Chionochloa pallens Tubulin (Tub) - MN106004
Chionochloa pallens Nucleic acid binding protein (NBP) - MN106005
Chionochloa pallens Expressed protein (ExP) - MN106006
Chionochloa pallens Tumour homolog protein (THP) - MN106007

Difference in the *CO* fold change relative to 18S normalisation in *Celmisia*

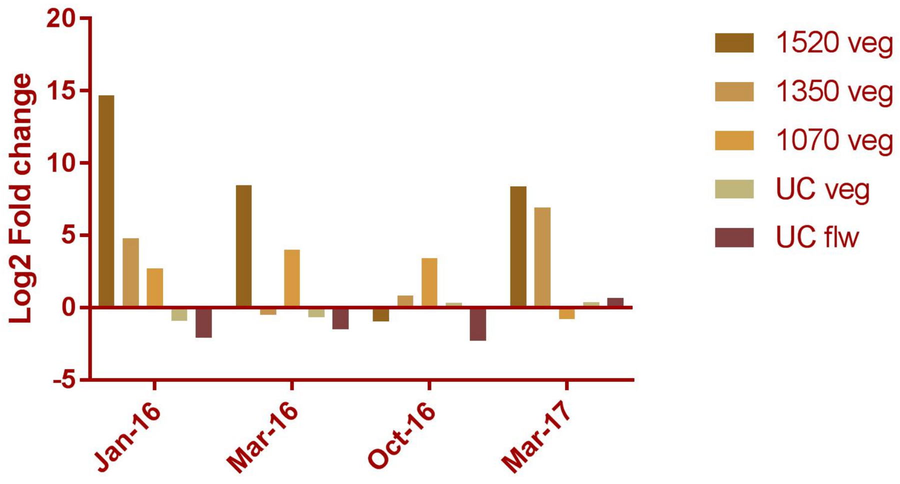

Difference in the *Hd1* fold change relative to 18S normalisation in *Chionochloa*

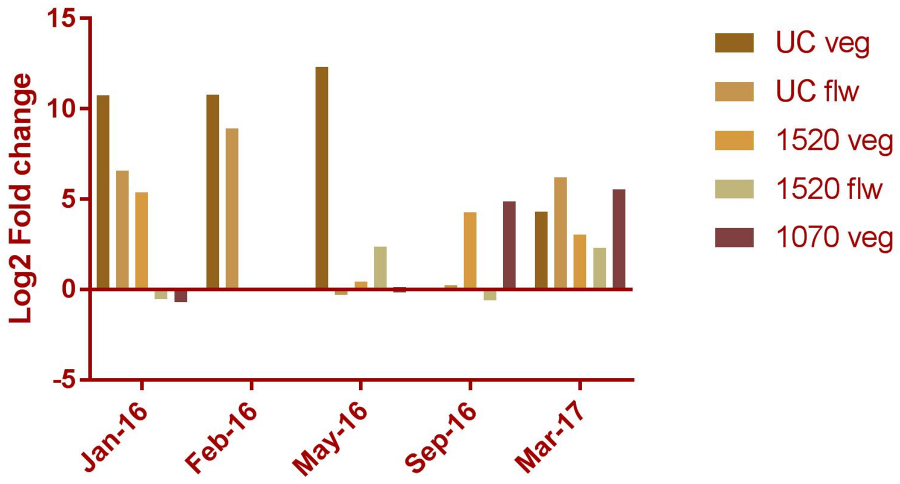

Supplementary fig S7. Differential $\log_2$ fold change in the expression levels of *CO* and *Hd1* when normalised with U18S reference gene relative to the expression levels of *CO* and *Hd1* normalised with selected best pair of reference gene. The huge change in the expression levels indicate how improper validation of reference genes can introduce biased errors in the gene expression data.
